# Hotspots of Large Rare Deletions in the Human Genome

**DOI:** 10.1371/journal.pone.0009401

**Published:** 2010-02-25

**Authors:** W. Edward C. Bradley, John V. Raelson, Daniel Y. Dubois, Éric Godin, Hélène Fournier, Charles Privé, René Allard, Vadym Pinchuk, Micheline Lapalme, René J. A. Paulussen, Abdelmajid Belouchi

**Affiliations:** 1 Department of Medicine, Université de Montréal, Montréal, Quebec, Canada; 2 Genizon Biosciences, Inc., St. Laurent, Quebec, Canada; Health Canada, Canada

## Abstract

**Background:**

We have examined the genomic distribution of large rare autosomal deletions in a sample of 440 parent-parent-child trios from the Quebec founder population (QFP) which was recruited for a study of Attention Deficit Hyperactivity Disorder.

**Methodology/Principal Findings:**

DNA isolated from blood was genotyped on Illumina Hap300 arrays. PennCNV combined with visual evaluation of images generated by the Beadstudio program was used to determine deletion boundary definition of sufficient precision to discern independent events, with near-perfect concordance between parent and child in about 98% of the 399 events detected in the offspring; the remaining 7 deletions were considered *de novo*. We defined several genomic regions of very high deletion frequency (‘hotspots’), usually of 0.4–0.6 Mb in length where independent rare deletions were found at frequencies of up to 100 fold higher than the average for the genome as a whole. Five of the 7 *de novo* deletions were in these hotspots. The same hotspots were also observed in three other studies on members of the QFP, those with schizophrenia, with endometriosis and those from a longevity cohort.

**Conclusions/Significance:**

Nine of the 13 hotspots carry one gene (7 of which are very long), while the rest contain no known genes. All nine genes have been implicated in disease. The patterns of exon deletions support the proposed roles for some of these genes in human disease, such as *NRXN1* and *PARKIN*, and suggest limited roles or no role at all, for others, including *MACROD2* and *CTNNA3*. Our results also offer an alternative interpretation for the observations of deletions in tumors which have been proposed as reflecting tumor-suppressive activity of genes in these hotspots.

## Introduction

Recent improvements in microarray-based genotyping technology have led to significant advances in our understanding of the genetic contribution to common disease in the last few years. In addition to identification of chromosome regions carrying haplotypes putatively involved in conferring disease susceptibility, these studies have also allowed quantitative assessment of large-scale deletions and duplications (also known as copy number variations, CNVs) on a genome-wide basis. The number of individuals carrying a given CNV at a known gene locus is frequently small, so conventional association studies based on statistical analyses cannot always be performed. Nevertheless, discovery of these anecdotal changes can be useful since, for example, deletion of even a portion of one copy of a gene expected to have an important biological role can have important consequences and can ultimately lead to insights into disease etiology.

A number of publications have documented copy number variations (CNV) in a variety of population samples using data from genome-wide association studies. These contributions have revealed potentially important roles for CNVs in disorders such as autism, schizophrenia and other neurological conditions [1]–[4]. In particular, a recurring observation is that of deletions in the very large neurexin-1 gene, and to these findings can be added the description of chromosomal rearrangements in or near the *NRXN1* locus in two subjects with autism spectrum disorder [5],[6]. Whether these changes are indeed contributory to disease, however, has been questioned in a report of a large autism study in which segregation of the deletions with disease was not observed in 4 of 6 families [7]. Resolution of this issue would be very instructive, as the gene in question is an important component of synapse complexes.

Genizon Biosciences has recently used the Illumina Infinium Hap300 platform to complete genome-wide studies of 550 attention-deficit hyperactivity disorder (ADHD) trios, 540 endometriosis (EN) simplex patients and 480 schizophrenia (SZ) simplex patients, as well as 640 recruited in a longevity study (LG; this sample comprised individuals more than 95 years of age and was genotyped on the Illumina Hap550 array). As a first step in determining the possible role of rare autosomal deletions in our samples, we evaluated fluorescence intensities as presented by the Beadstudio program, using a combination of computer-based scanning and individual assessment by human observer. The trio structure of the ADHD study allowed mutual verification of each transmitted deletion in both parent and child, and we were able precisely to define those SNPs situated within each deletion. We found remarkable patterns of clustering, with variation in frequency of independent deletion events per unit length of as much as 100-fold across the genome. The points of greatest deletion frequency fell within about 15 regions mostly of about 0.4–0.6 Mb in length; 13 of these were evaluated in the other cohorts and found to be similarly enriched for deletions. Four of the regions had no genes whereas nine harbored a single gene or gene region, all nine of which have been implicated in disease. The patterns of exon loss vs. retention allowed insights into the role, or lack thereof, of the genes in the respective diseases.

## Results

The number of transmitted and non-transmitted autosomal deletions in the ADHD trios: The analysis of the offspring as described in the Methods and in the Supporting Information S1 resulted in 343 rare, independent candidate deletions in 440 cases which were called by PennCNV and supported by visual inspection. These are referred to as affirmed deletions. 23 of these were also present in a second individual for a total of 366. Of these, 274 had the same deletion called by PennCNV in one or the other parent. Visual inspection of the corresponding Beadstudio images showed that 85 of the remaining 92 were false negatives, in that identical deletions were clearly demonstrable in one of the parents. A total of 359 transmitted deletions (336 independent) were therefore found in this round, and we conclude that 7 of the affirmed calls were *de novo* deletions.

In the second round we examined the deletions called by PennCNV in the parents, and found 549 independent rare deletions which were visually affirmed, of which 49 were present in a second parental sample for a total of 598. Of these 598, 270 had called, affirmed deletions in their offspring from the first round. The samples of the offspring of each of the other 328 parents were visually examined in the Beadstudio application for any uncalled deletions corresponding to that found in the parent. Thirty-three were found, of which 6 had already been found in other trios. (Possible reasons for the lower rate of apparent false negatives in the second round are discussed in Supporting Information S1.)

This iterative process therefore revealed a total of 399 deletions, counting the *de novo* events, in offspring for both rounds of inspection. 368 were independent (listed in the first table of Supporting Information S1), and since we calculate the sensitivity of this process to be about 0.94 (see Supporting Information S1), they probably represent nearly all of the deletions in the offspring that are detectable with this approach. Among the non-transmitted chromosomes we detected 270 different affirmed deletions, of which 20 were present in a second non-transmitted chromosome, for a total of 291 detected in all non-transmitted chromosomes. This number is almost certainly less than the real number, since the sensitivity of PennCNV without the benefit of the 2-generation cross-verification is estimated at about 0.75 (see Supporting Information S1). Indeed, the respective calculated sensitivities suggest that the numbers in the transmitted and non-transmitted chromosomes were not significantly different, at about 390 and 360 respectively.

**Figure 1:**
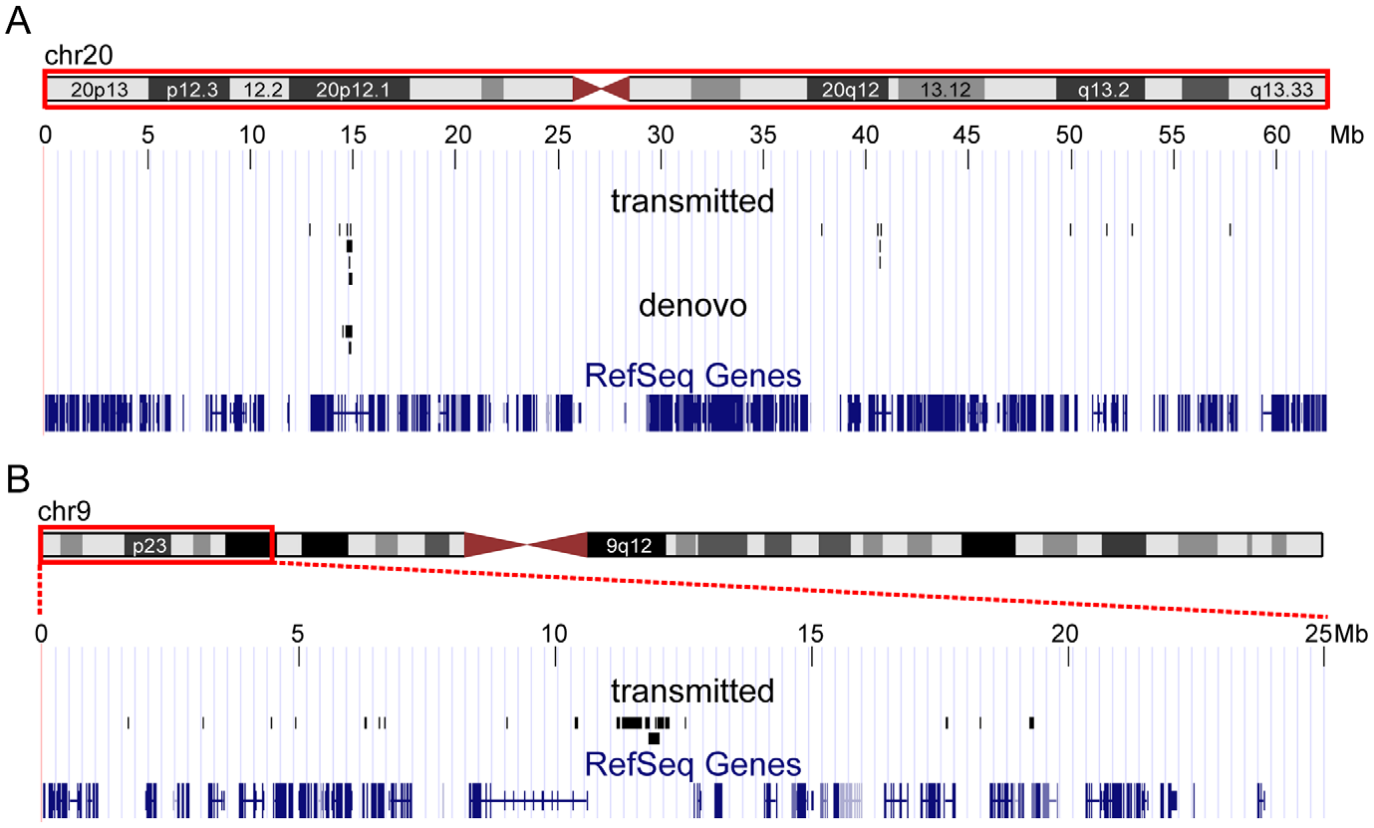
Distribution of transmitted and *de novo* deletions in the ADHD sample of 440 parent-parent trios. Two regions with particularly high frequency of deletions are presented, using the UCSC Genome Browser. A. Chromosome 20. The hotspot in 20p12 was the most unstable, with 27 independent deletions in four population samples comprising 2540 individuals. A second hotspot was observed on this chromosome, at about 40.6 Mb. B. First 25 Mb of Chromosome 9. No *de novo* deletions were documented in this chromosome.

Distribution of the deletions in the genome: A striking observation is that many of the deletions cluster within a limited number of chromosomal regions. Examples are shown in Fig. 1A, which illustrates the results for all of chromosome 20 on which two clusters were found. Of the total of 16 transmitted and 3 *de novo* deletions found in the 440 ADHD patients, all of the *de novo* and 5 of the transmitted deletions were located between 14.5 and 15.0 Mb, and a further 4 transmitted deletions were found in another region at approximately 40.5–40.7 Mb. Fig. 1B shows the first 25 Mb of chromosome 9, carrying a cluster of 5 deletions in band 9p23. (Note there is one large deletion immediately before this cluster which could arguably be considered as being within the region.)

Similar deviation from uniform distribution was seen in several other regions, which generally ranged from 0.4–0.6 Mb in length (Table 1). For purposes of calculation and discussion, we choose the unit length of these regions, which we refer to as ‘hotspots’, to be 0.6 Mb, since some of the apparently shorter domains, such as that at 40.5–40.7 Mb on Chr20, may in fact be longer than shown in Table 1 simply because not enough deletions have been found to represent the full length of the region. A Poisson distribution analysis (Supporting Information S1) revealed that, assuming no bias in distribution of deletions, the number of 0.6 Mb chromosome segments carrying 2, 3, and 4 independent rare transmitted or *de novo* deletions is expected to be 14, 0.38 and 0.008, respectively, whereas the numbers actually found were 33, 6 and 5. The two domains with 5 and 8 deletions were even further removed from the pattern predicted by the Poisson equation if no bias existed, with about 10^−4^ and 10^−10^ expected, respectively. We can therefore state with confidence that virtually all 13 domains with 3 or more deletions were unexpectedly ‘hot’. Furthermore, since 19 more domains than expected were found in which two deletions resided, we interpret the results as suggesting that the real number of hotspots may be of the order of 30 or more. This in fact represents a minimum, since we have no way of knowing how many hotspots may exist for which a deletion is lethal, or results in a phenotype excluded by our recruiting criteria. (This is illustrated by data presented below, where we find, as have others, that the hotspot in 2p16.3 is preferentially deleted in SZ patients

**Table 1:** 13 genomic regions with high frequency of rare deletions in four population samples.

			Number of independent deletions found (number of deletions affecting coding exons)					
			ADHD			EN	SZ	LG
Region location	Limits of region (Mb)	Gene	Trans-mitted*	non-transmitted	Independent in all chromosomes			
1q43	235.2–235.7	none	4 (na)	0 (na)	4	1 (na)	1 (na)	1 (na)
2p16.3	50.7–51.3	*NRXN1*	2 (1)	1 (0)	3	2 (0)	7 (2)	3 (0)
6q26	162.5–162.9	*PARKIN*	4 (2)	5 (4)	9	1 (0)	3 (2)	6 (1)
8p23.2	4.3–4.9	*CSMD1*	3 (0)	1 (0)	3	3 (0)	2 (0)	3 (0)
8p23.2	5.6–6.2	none	2 (na)	2 (na)	4	3 (na)	3 (na)	1 (na)
8p22	15.2–15.6	*TUSC3*	4 (1)	1 (0)	5	0 (0)	0 (0)	1 (0)
9p23	11.7–12.2	none	5 (na)	5 (na)	9	7 (na)	8 (na)	13 (na)
9p21.1	30.4–30.7	none	3 (na)	0 (na)	3	1 (na)	3 (na)	1 (na)
10q21.3	67.8–68.2	*CTNNA3*	3 (3)	5 (4)	7	8 (4)	4 (2)	8 (4)
13q31.1	83.1–83.7	*SLITRK1*	4 (0)	4 (0)	8	2 (0)	2 (0)	3 (0)
16p13.2	6.6–7.0	*A2BP1*	0 (0)	4 (0)	4	1 (0)	4 (1)	4 (1)
20p12.1	14.5–15.1	*MACROD2*	8 (0)	4 (0)	11	9 (3)	6 (0)	3 (0)
20q12	40.5–40.7	*PTPRT*	4(0)	0 (0)	4	2 (0)	0 (0)	1 (0)

ADHD, attention deficit hyperactivity disorder, 440 parent-parent trios; EN, 540 endometriosis simplex patients; SZ, 480 schizophrenia simplex patients; LG, 640 individuals over 95 years of age.

*includes 4 de novo deletions, three in 20p12.1 and one in 2p16.3.

The genomic distribution of deletions in the non-transmitted chromosomes was then examined and again these were found to cluster in hotspot regions. Eight of the 11 loci in the ‘transmitted’ list of hotspots were also in the list of non-transmitted deletion hotspots, and only one locus (chr16p13.2) with no transmitted deletions was represented on the non-transmitted list of hotspots (Table 1). On the other hand, those deletions in the bulk of the genome (‘non-hotspot deletions’) were distributed in an apparently random fashion. The average enrichment for deletions per unit length of DNA in the hotspots was about 50-fold in both transmitted and non-transmitted chromosomes (Table 1), rising to more than 100-fold in the most extreme cases of 9p23 and 20p12.

To determine the universality of these hotspot domains, 13 of the domains with three or more deletions in the entire ADHD study were assessed for deletions present in other samples. The PennCNV algorithm was used to identify candidate deletions within Genizon's SZ and EN samples, and subsequently the visual inspection protocol developed for ADHD was used to confirm these loci (Supporting Information S1). Whereas the overall density of affirmed non-hotspot deletions was about the same in the other cohorts as for ADHD (with the exception of SZ which was about 50% higher; ref 4 and our results not shown), we again observed clustering in the same regions as in the ADHD trios, indicating that the clustering effect was not limited to this cohort (Table 1).

A third sample for longevity, which had been genotyped with the Illumina 550k microarray was analyzed separately and again, clustering was observed within the same hotspots (Table 1).

### Characteristics of the Deletions and the Hotspots

The mean length of the 135 different clustered deletions which were found in all samples was 94 kb, very close to the mean for the non-HS transmitted ADHD deletions, but the median was 54 kb, nearly twice that of the latter. The standard deviation was much greater in the latter group, (219 kb vs. 111 kb). These numbers reflect a greater uniformity in length in deletions found in hotspots, with 68% being between 20 and 200 kb, compared with 50.2% for non-HS deletions (chi square 4.01, p<0.05) falling within this size range. This length corresponds very approximately with the length of chromatin loops, and may reflect some aspect of the mechanism for this high frequency deletion phenomenon.

We were able to trace the parent-of-origin for five of the *de novo* deletions, being the hotspot deletions in 2p16.3, 6q26 and 20p12.1. In three of the five, the deletion was maternally inherited, a ratio similar to that found in the major hotspot in the DMD gene (P. Helderman and E. Bakker, personal communication).

With respect to gene content, a remarkable characteristic of the hotspots is the length of the genes therein. Nine of the domains have one and only one gene, and 7 of them rank among the 35 longest genes in the genome (according to gene annotations in the UCSC Genome Browser), including 4 in the top 8. The regions of greatest deletion frequency are in the regions of very long introns in the long genes, usually the 5′ end. Interestingly, the DMD hotspot extending from exon 44 to exon 53 which is comparable to those described here is in the longest gene. In any event, it is clear that the coding sequence density is, on average, very low in the hotspots. As noted below, none of the sequence motifs, or functional entities known or suspected to be involved in chromosomal instability are present in abundance in any of the hotspots. Furthermore, the proximity of fragile sites, which overlap with only one of the 13 regions we identify (6q26), does not explain the major part of this instability.

## Discussion

We have documented the presence of 13 regions of about 0.5 Mb each in the human genome where deletions occur at up to 100-fold higher frequency than the other 99.8% of the genome. We also present suggestive evidence for the existence of as many as 30 or more hotspots throughout the genome (Poisson distribution, Supporting Information S1). With the exception of 2p16.3 (the *NRXN1* gene) we found approximately equal numbers in all samples, and we present (below) indications from the published literature that the hotspots are found in populations other than the Quebec Founder Population; therefore their existence is probably a universal phenomenon.

By filtering out frequently observed deletions, we eliminate from consideration common deletions existing in the population (by analogy with SNPs, probably those which arose a relatively long time ago and against which there is no negative selection) as well as those rare but recurring deletions arising between repeated elements such as segmental duplications. In so doing, we maximized the chance of finding regions where deletions frequently occur due to reasons other than the presence of repeated elements, such as those in 16p11.2 associated with autism [7].

**Figure 2:**
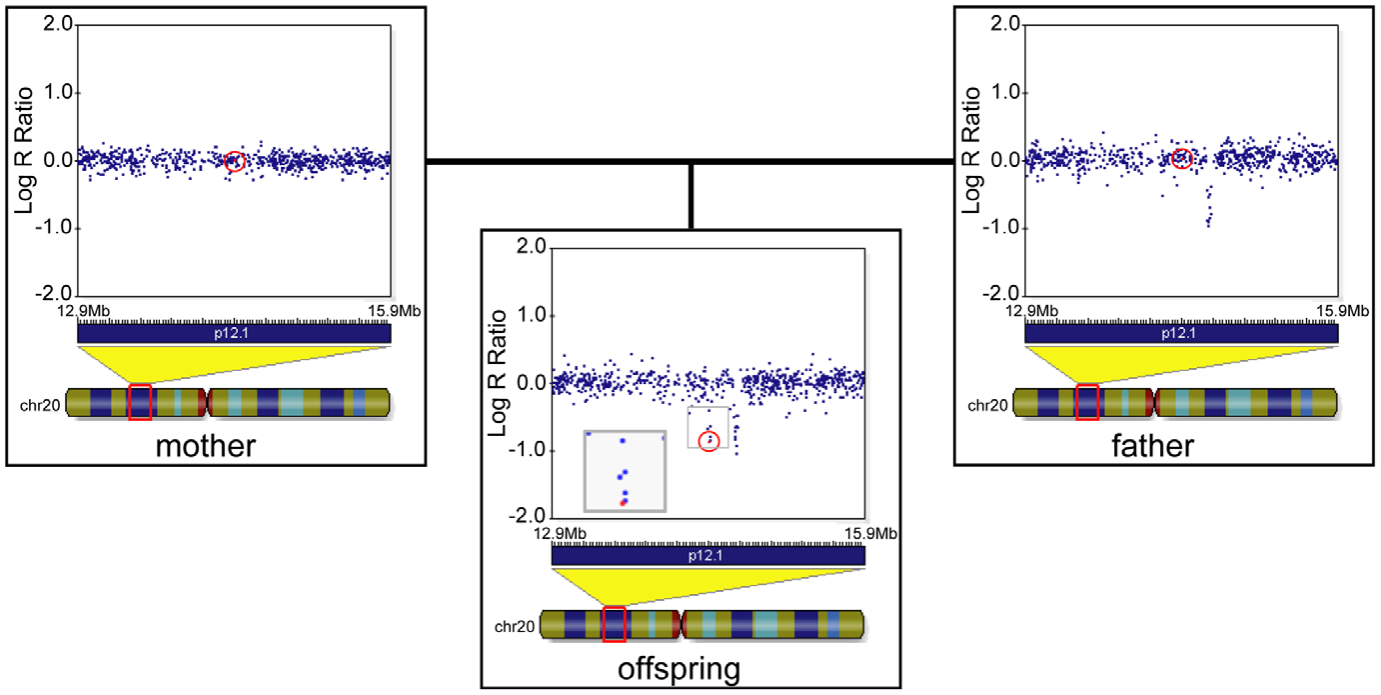
Illumina Genome Viewer display of LogR ratios in the hotspot region of 20p12 in one trio. The offspring shows an inherited deletion from the father, as well as a *de novo* deletion occurring adjacent to the former. The same SNP is circled in each panel.

The hotspot in 20p12.1 is particularly noteworthy. A total of 27 independent deletions were documented in the 4 population samples, 25 of which were seen only once. The exactitude of the deletion borders is not in question, as the log R ratio graphs show how cleanly the first and last SNP of each deletion could be called (illustrated in Supporting Information S1). The instability of the region is underlined by the detection of 3 *de novo* deletions within the 440 ADHD children, one of which is adjacent to another deletion inherited from the father (Figure 2).

Although this is to our knowledge the first such genome-wide description of genomic instability at this level, our conclusions are supported by published work in a variety of ways. First, deletions in two of the genes residing in hotspots have been intensively studied by many groups because of their roles in disease. Deletions in the *PARKIN* gene (Chr6q26) are involved in about one-half of familial early-onset Parkinson's disease (PD) cases; as reviewed by Hedrich et al. [8] exons 3 and/or 4 (corresponding to the hotspot in 6q26) are deleted in 50% of cases, whereas exons 1 and 10, farthest from the hotspot we identify, contribute less than 2% to the total of exon deletion events. Similarly, more than 90% of the 66 deletions found in the 1.1 Mb *NRXN1* region of Chromosome 2 by Rujescu et al. [9] cluster in the same 0.5 Mb where we have identified deletions.

Second, in a genome-wide study similar to ours [10] several hundred heterozygous deletions were detected in 810 individuals, at about the same frequency as in the present study. When recurring deletions were removed and the remainder analyzed for clustering (our data-handling, not shown) patterns very similar to ours are obtained, in almost exactly the same regions, especially in 20p12.1, 9p23 and 10q21.3 (6 or 7 independent deletions each). Third, the DGV database (http://projects.tcag.ca/variation/; UCSC Genome Browser) typically documents many deletions at the hotspot sites we have identified. The inevitable imprecision in mapping deletion endpoints when using the available CNV-calling algorithms [11] makes it difficult to assess whether they represent independent events, rather than simply the same common deletion detected with variable apparent endpoints. Nevertheless it is probable that the high frequency of detection of these events reflects the hotspot nature of the genomic domain in at least some instances. Finally, as discussed below, deletions are found in primary tumors as well as tumor-derived cell lines in several of the hotspots we define here, notably 20p12 [12], 6q26 [13] 10q21 [14].

It is of interest to place these findings in the context of what is known regarding hotspots of chromatin instability, and deletions in particular. One of the few such regions which are well enough characterized to allow estimates of deletion frequency is in the *DMD* gene, which at about 2.4 Mb is the longest known gene (this was not formally included in our study since it is not autosomal). The incidence of Duchenne muscular dystrophy is 1 per 3500 males and it is known that 1/3 of the cases are attributable to *de novo* mutations, of which 60% are deletions [15]. A further 6% are duplications, which we are not considering for this discussion. Deletions arising in the major hotspot, involving introns 40–54 and covering 0.7 Mb (about the same as the length of our hotspots), comprise about 2/3 of all those known in DMD. This suggests a frequency of ascertainable *de novo* events of approximately 4×10^−5^ per 0.7 Mb. A number of deletions presumably occur in this region which do not affect exons, and have therefore never been ascertained. Given that the median deletion length and median intron length in this region are respectively about 60 kb and 36 kb, we may project the total frequency of deletions which lie entirely within introns as somewhat less than equal to the frequency of ascertainable deletions which knock out an exon. Thus we conservatively project a *de novo* deletion frequency of 8×10^−5^ per generation per chromosome per 0.7 Mb in the hotspot.

In our study, we observed 5 *de novo* deletions in a total of 13 hotspots covering about 7 Mb in 440 individuals. If representative, this number reflects a rate of about 4×10^−4^ per generation per chromosome per 0.6 Mb (two parental chromosomes can contribute to a deletion in autosomal loci). This is at least five times more frequent than the major hotspot in DMD, and 2 or 3 orders of magnitude higher than the remaining 99.7% of the genome, where only 2 *de novo* events were detected by us. (We project this number to represent 4 events in total because of the false negative call rate; see SI.) In the most active hotspot (Chr20p12.1 where we found 3 *de novo* events), deletions occur at about 10 times the rate seen even in intron 49 of the DMD gene, where the greatest density of deletion clustering is seen. Therefore we consider that at least some of the hotspots we describe are considerably more unstable than any which have been quantitatively defined to date. Consistent with this is the detection of only one deletion in the *DMD* gene hotspot in any of our 2540 individuals (exon 48 in an individual in the EN sample; results not presented) or the 810 studied by Blauw et al. [10].

This work raises a number of intriguing questions at the fundamental level, one being, why do these hotspots exist? It may be that as a consequence of some form of stress, a chromatin loop may escape its natural confines within the highly organized and compact nuclear structure, and this event simply happens much more often at these sites. Alternatively, these high-frequency deletions may reflect some protective element, for which positive selection has occurred. It is of note that these scenarios are not mutually exclusive, in that there may exist situations of stress where a chromatin domain may (or must) undergo deletion; it would be to the organism's advantage if the deletion occurred in a DNA domain of low coding sequence density. In this way the hotspots we have characterized could be considered as hypothetical safety valves.

A second question that can be raised concerns the molecular mechanism of the high frequency of deletions. Many of the chromosomal elements such as low copy repeats (LCR), and segmental duplications (SD) which have been associated with structural alterations identified in diseases such as autism, neurofibromatosis and Sotos syndrome (OMIM) have been ruled out in the case of the DMD hotspot [15] and recently in the chr6q26 hotspot [16]. Likewise, upon initial analysis we have found no particular clustering of any of these with breakpoint hotspots in our collection. Hotspots of recombination, invoked to explain some deletion patterns, are spread across the genome at intervals of ten to hundreds of kb (as visualized in the UCSC Genome Browser), and although they may be the preferred site of breakpoints when structural alterations in specific genes lead to an identifiable phenotype, it is difficult to see how the presence of tens of thousands of these sites may explain the existence of the handful of deletion hotspots we have identified. Similarly, fragile sites appear not to be associated in a significant way, in that only two hotspot regions, 6q26 and 8p22 are in bands with fragile sites, FRA6E and FRA8B respectively, based on the summary of 113 sites in about 310 bands by Calin et al. [17], and the latter probably does not overlap the hotspot. The only finding which may be pertinent to this discussion is the report of increased incidence of double strand breaks in intron 49 in the DMD hotspot when transfected into yeast [15]. This may reflect, for example, increased TOPO2 activity, but at present we have no evidence to implicate such activity in any of the regions in question.

At a more applied level, these data also have implications for gene-disease associations. The finding of rare deletions in or near coding sequences, especially if they arise *de novo* in probands, has often been accepted as *de facto* evidence that the affected gene may be involved in the condition in question, simply because of the expected low frequency of these events in the genome (examples below). Our findings indicate that this argument does not hold for deletions occurring in the hotspots we have documented, and since the Poisson distribution analysis (Supporting Information S1) indicates other hotspots exist, this is probably also true for a number of other regions.

Nine of the documented hotspots carry genes, and every one has been implicated in disease. We propose that a careful delineation of precise deletion (or amplification) boundaries in and around these genes will be useful, since at least some of the deletions may be present simply due to the unstable nature of the chromosomal domain rather than because they contributed to the phenotype by affecting gene function. In our samples, exons were unaffected in three of the nine genes, perhaps reflecting important roles for these genes in human health; however because of small numbers involved we cannot draw conclusions from this information. Nevertheless, the patterns of exon disruption in the other genes are somewhat informative, and the following paragraphs present some examples.

### *NRXN1* and Autism

Deletions in this gene have been implicated in neurological disorders including autism and mental retardation in anecdotal fashion [1]–[4]. A major family study of autism [7], on the other hand, found deletions which did not segregate with the condition, and the authors concluded that there was no association. We suggest that a close assessment of exon dosage in these families may reveal either association, or the lack of it, between deleterious deletions and autism. One may postulate that the majority of the deletions in this region segregating in the families do not affect coding sequence, and their presence reflects merely the hotspot nature of the domain; those few which actually disrupt exons may be shown to segregate with the condition. This scenario could be predicted based on our results: 2 of 7 deletions in the SZ sample affected coding regions of the gene, and one (*de novo*) deleted an exon in an ADHD proband. None of the five deletions found in other samples (EN, LG) affected exons. Similarly, a recent large study assessing CNVs in *NRXN1* in more than 35,000 individuals [9] found CNVs in the SZ group at a frequency 3 times higher than amongst controls, but in both patients and controls most CNVs did not affect exons.

### *MACROD2* and Kabuki Syndrome

One report suggests this gene as a candidate for Kabuki syndrome, since a *de novo* deletion involving exon 5 of this gene was found in a proband [18]. Our finding of three individuals from the EN cohort with deletions of exons 5 and/or 6 reduces the likelihood of this proposed association being real, as a review of the files of each of the 3 individuals showed no Kabuki-like symptoms at all. The location of the gene in a hotspot of deletion greatly increases the chance of sporadic exon deletions, and perhaps explains the chance finding of the deletion in the Kabuki proband. On the other hand, the incidence of exon-deleting mutations in the EN cohort (3 out of 9) compared to 0 of about 16 in the other cohorts suggests a possible involvement of this gene in EN.

This region of the genome has also been implicated in colorectal cancer, with the report [12] that 23% of primary tumors and 55% of cell lines had undergone deletion events with the consensus minimum region of loss at 14.85–15.05 Mb, coinciding with the hotspot we defined here. This group provided evidence that RNA molecules encoded in the region may have tumour suppressor activity, but it is also probable that the high frequency of deletions may in part be attributable to the instability of the region.

### *CTNNA3* and Alzheimer's Disease

The hotspot on chromosome 10 falls in the 3′ half of this gene. Exons were affected in a substantial proportion of the deletions, including four such deletions which would produce a frameshift in the LG cohort (all 4 subjects were mentally alert). This gene has been associated with late-onset Alzheimer's disease in women by genetic studies [19]. The results reported here suggest that if *CTNNA3* is involved in Alzheimer's it is not through a loss-of-function mechanism.

### *PARK2*/*PARKIN* and PD

The association of this gene with familial early-onset PD is well established, since it is homozygously mutated in about 50% of such cases [8]. The gene is mutated in a proportion of later-onset PD, but it is currently uncertain whether single-copy deletions in fact predispose to this condition. Our results may be pertinent in this debate. Of the 2540 unrelated individuals in our studies, 22 (0.8%) carry deletions in this gene; exons are affected by 10/15 of the deletions in the ADHD, EN and SZ samples (virtually all of whom are under the usual age of PD onset), but only by one of the 6 deletions in the LG sample, none of whom had PD in spite of advanced age (p<0.05). These results are not inconsistent with a role for *PARKIN* deletions in late onset PD and follow-up of patients carrying deleterious deletions like those we have found may help resolve this issue.

### Cancer

One of the hallmarks of a tumor-suppressor gene (TSG) is the presence of deletions in tumors which affect coding sequence, as was seen with the prototypical TSG, RB1 [reviewed in 20]. If the deletion is inherited, the classic pattern observed is the formation of multiple tumors in the susceptible tissue, since each cell is in principle predisposed to cancer. The search for deletions in tumors has produced many TSG candidates, and some of the sites frequently reported coincide with the hotspots described here. In particular, a paradox has arisen in the case of the *PARKIN* gene which our data may help resolve. This gene is described by some groups as a TSG mainly on the strength of the frequency of exon-disrupting deletions in cancer [13], [21] but patients with PD appear to be, if anything, protected from most cancers [22]. In the extreme situation of individuals inheriting two mutated alleles (engendering early-onset Parkinson's disease), one would expect the appearance of multiple tumors, an observation which has not been reported. However, if deletions occur at high frequency in certain chromosomal sites in cancer merely as a consequence of the unstable nature of the chromatin domain, their appearance would not justify attributing tumor-suppressive function to the gene product. Similar arguments apply to the very high frequency of deletions in Chr20p21.1 in colorectal cancer, which overlap between 14.85–15.05 Mb [12], in the most active hotspot we have found. *CTNNA3* [15] and *TUSC3* [23], which have also been cited as candidate TSGs for the same reason, could also have their status as TSG re-evaluated in light of our results, given that for each of these genes we have identified a number of cancer-free adult individuals with exon-disrupting deletions (Table 1).

In general, therefore, the existence of hotspots with the properties we present here should be incorporated into any interpretation of deletion data concerning the genes associated with these hotspots. In some instances, it may become appropriate to incorporate exon-dosage assays in evaluating individuals' risk and potential treatment scenarios.

## Methods

Ethical approval was obtained from Ethica, Montreal, for all stages of recruitment and data generation [24] and all subjects gave signed Informed Consent. Sample collection and phenotyping were performed according to Genizon protocols as has been described [24]. DNA was extracted from the buffy coats of blood samples taken from donors and genotyped on Illumina Infinium HumanHAP300 arrays (HumanHAP550 array in the case of the longevity sample) according to the manufacturer's instructions.

PennCNV [11] was run on the Illumina Beadstudio data according to the creators' instructions. For the ADHD sample, after removal of incomplete trios and trios with high variance in the log R ratios (generating more than 20 deletion calls by PennCNV per individual; see Supporting Information S1), 440 trios were available for analysis. The results were processed as described in the Supporting Information S1 to eliminate all but the rare deletions (defined below).

Determining CNV boundaries with precision is a significant issue in calling structural variations in the genome [11]; see also Supporting Information S1. When the creators of PennCNV tested the boundary precision by comparing inherited CNVs in offspring with those in the parents [11] many were found to be inaccurately called. We reasoned that combining PennCNV with visual inspection of Beadstudio images might improve the precision of CNV calls. Samples with a copy number of 1 at a given SNP should show up as points of reduced but non-zero normalized R value, hence below the main cluster as illustrated in Figure 3b of [reference 24] and in the Supporting Information S1. As discussed in Supporting Information S1, we chose a minimum of 3 contiguous SNPs at low normalized R value as representing a deletion event. Several deletions were selected for Syber Green qPCR, with 100% concordance between called deletions and PCR results (Supporting Information S1).

The ultimate goal of this work was to detect genomic deletions potentially associated with disease, so we anticipated that the pertinent alterations might be rare, as has been found for schizophrenia (SZ) [2], [4]. Consequently we restricted our evaluations to deletions occurring in only 1 or 2 individuals per sample of about 500; in so doing, the complexity of the Beadstudio clusters to be analyzed was reduced to a level at which visual inspection became feasible (see Supporting Information S1).

## Supporting Information

Supporting Information S1 Text, figures and tables presenting information not shown in the main text.
(3.61 MB DOC)
