# Supplementary material for: Hotspots of Large Rare Deletions in the Human Genome
Source: PLoS One. 2010 Feb 25;5(2):e9401. doi: 10.1371/journal.pone.0009401 (PMC2828468; doi:10.1371/journal.pone.0009401)
Supplement: Supporting Information S1 — Text, figures and tables presenting information not shown in the main text. (3.61 MB DOC) [file pone.0009401.s001.doc]

**Supporting Information S1**

**The Nature of low Normalized R values**

As described by McCarroll et al. [1], the visual presentation of fluorescence intensities (Normalized R values) of the clustered data points in the Illumina BeadStudio can reflect the DNA copy number for each DNA sample at each of the 317,000 SNPs in the Infinium HumanHAP300 microarray with near-zero and intermediate R-values potentially representing, respectively, homozygous and hemizygous deletions. Since the objective of this project depended on using these intermediate R values to identify deleted chromosome regions, we initially sought to determine the molecular basis of low fluorescence data points. We reasoned that this would best be done by analyzing the DNA at points where a given single SNP yielded an R value of zero (or near-zero) whereas the adjacent SNPs gave normal R values but the genotypes were homozygous over a significant distance in this region. In this way the molecular basis for lack of fluorescence would be the same for both alleles, so analysis would be simplified. Twenty-five isolated SNPs were identified at random in 25 different samples which yielded near-zero R values in BeadStudio such that the same sample was homozygous (but of average R value) for at least 30 SNPs upstream and downstream. These were assumed to be autozygous, i.e., the two alleles were identical by descent. PCR amplification of the 300-500bp around the near-zero SNP was attempted for each of the 25 samples, and for 23 we succeeded, indicating that 2 were probably authentic homozygous deletions. Fourteen of the 23 amplified bands were sequenced, and all yielded the expected sequence with the exception of a single variation in 13 of the samples between 1 and 5 bases from the target SNP, and for the 14^th^, 2 variations 6 and 15 bases away (data not presented). We concluded that the majority of isolated low fluorescence readings were not due to deletions, but to reduced hybridization strength resulting from the mismatch. A scan of chromosome 1 revealed that only about 20 of 23,275 SNPs had near-zero readings (i.e. were probably homozygous for a nearby interfering SNP), so the frequency of nearby confounding SNPs is very low. We accordingly chose a run of 3 SNPs with low R values as the minimum length to indicate the presence of a deletion.

**Evaluation and validation of candidate rare deletions**

PennCNV is a freely available Hidden Markov Model [2] that identifies CNVs in datasets generated by any of a number of genotyping platforms. The creators of the program concluded that the Illumina platform was superior to the other platforms tested with respect to fine definition of small CNVs, with median sizes of 13kb on the HumanHap 550 array vs. 81 and 204 for the Affymetrics 500_EA and the Whole Genome Tilepath Array, respectively [2]. They also found that a significant issue in calling structural variations in the genome was the precision of the boundaries of the CNV calls. The authors tested PennCNV using family information from HapMap trios and found it to be superior in this respect to another available program. However when they tested boundary precision by comparing inherited CNVs in offspring with those in the parents they found that many CNVs were not accurately called. We reasoned that combining PennCNV with visual inspection of BeadStudio images might improve the precision of CNV calls. Samples with a copy number of 0 or 1 at a given SNP should show up as points of reduced normalized R value, hence below the main cluster as illustrated in Fig. 3b of ref. 1.

We reasoned that a given individual in the cohort would not have a significantly different likelihood of carrying genomic deletions than the cohort as a whole; accordingly, in a first step, PennCNV calls of copy number 1 and with log Bayes above 12.0 (the minimum reliable value as we have found, data not shown) were treated in an Excel file to allow removal of samples carrying 20 or more apparent deletions, assuming the hybridizations for these samples gave R values which were too variable. This was confirmed by observation of a much greater degree of scattering of the log R ratios in Genome Viewer for these DNA samples. The remaining entries were listed by SNP number and series of more than 4 entries with identical SNP number in either cases or controls, and which had similar log Bayes values (indicating they were the same length) were removed on the expectation that they were common deletions, as we define them.

**Visual inspection**

The candidate rare deletions, numbering about 1900, were then assessed by visual inspection of BeadStudio images. For each candidate deletion, all of the SNPs in the PennCNV-called deletion, plus about 10 further SNPs in each direction were evaluated for Normalized R values of the DNA sample in question falling below the cluster. We required at least one-third of a run of SNPs to yield data points below the cluster and most of the rest to be at the bottom of the cluster, as shown in Fig. 3b of ref. 1 and in Fig. S1. A source of potential confusion was the inclusion within an authentic run of SNPs with low Normalized R of a SNP with a nearby interfering SNP effect (see Nature of low R values, above). This could either falsely extend a run (if the sample carried the interfering allele and this was the SNP following or preceding a run) or give the appearance that the R value for a SNP within a run was not reduced (if the sample did not carry the minor allele of the interfering SNP, it would be buried in the midst of the points of samples carrying the interfering SNP).

Conversely, we considered that the appearance of even one heterozygous (AB) BeadStudio point clearly below the AB cluster occurring in or adjacent to a run of otherwise homozygous SNPs with low R values to be an indication of a probable false positive; similarly, a high R value in a run of homozygous SNPs with otherwise low R values was a reason to consider rejection of the PennCNV call. These questionable calls were then examined in the Genome Viewer tool of BeadStudio to evaluate the stability of the logR ratios over a 2-3mb region in the vicinity of the call. In virtually every case, a clear distinction was visible between points within the deletion and points corresponding to the diploid portions of the chromosome. Examples are shown in Fig. 2 (main text) and Fig. S2.

We refer to PennCNV calls which were supported through this procedure as *affirmed* calls.

We then performed a Poisson distribution analysis of the observed number of samples with 1, 2, 3,…n different putative deletions compared to the expected numbers. The DNA samples with 0, 1, 2, 3 and 4 apparent deletions each fit the Poisson distribution very closely; no samples were found with 5 or more affirmed deletions, indicating that as expected, these deletions were distributed randomly amongst the population sample.

**Specificity**

The data from the ADHD cohort presented the chance to measure the specificity of this procedure and to test the confidence level in our calls, since the parent-parent-child trio structure of this study allowed mutual verification of each transmitted deletion in both parent and child. Samples with PennCNV calls giving log Bayes values above 12 were visually evaluated with the BeadStudio application, without knowledge of kinship to other samples. About 50% of calls were clearly or probably false positives, using the criteria described above in ‘Visual Inspection’. All PennCNV calls in offspring, both those judged to be authentic (affirmed) and those judged to be false positives, were then compared to the respective set of parents. None of the false positive calls were found to have a parent with a BeadStudio pattern indicative of deletion at the sequence in question, whereas 98% of those affirmed by inspection were also present in one, and only one, parent, the only exceptions being 7 probable *de novo* events. In addition, the degree to which transmitted deletions in the two generations coincided yielded a further measure of confidence in the interpretation. This is illustrated by the group of runs of 3 SNPs in which would arguably be found the most questionable calls. 82 candidate deletions were in this group, and virtually all were as clear-cut as the examples shown in Figure S2A and B, where concordance is demonstrated between the offspring and parental samples for each SNP. Since these covered only 3 SNPs they would be expected to carry less assurance of authenticity than longer deletions, but both parent and offspring deletions clearly coincide perfectly, greatly increasing the level of confidence in the result. Among the 393 deletion calls which were found in both generations, the boundary definitions were very reproducible, with only one case of slight disagreement, which extended for three SNPs (data not shown). This may have simply been an outlier effect.

**Sensitivity**

Given the relatively high rate of PennCNV false negatives, we next wanted to approximate how many of the transmitted deletions we may have missed completely. We reasoned that these would comprise the group of those transmitted deletions not called in either parent or child which we had not already fortuitously found. The false negative rates in the parents and offspring were 86/(274+86) vs. 33/(270+33) or 0.239 vs. 0.109, respectively. The lower rate of finding false negative calls in the offspring (yielding the higher apparent sensitivity) may be a chance occurrence, or may reflect in part the painstaking nature of the search: it was expected that nearly all the deletions in the offspring would be transmitted, given the expected low rate of *de novo* events, whereas the majority of the deletions in parents which were not called in offspring by PennCNV were expected not to be found simply because they were not transmitted. The motivation to search for a pattern which is probably not present is less than the motivation to search when it almost certainly is present. In any event we assume conservatively that the former figure is more accurate. If the chance of missing either a parent or child is 0.239, the chance of missing both would be the square of this, 0.057, suggesting that only about 25 remained undetected in our cohort. If the assumption is correct, this corresponds to a sensitivity of 94%. The small number of samples missed is not included in subsequent calculations.

Thus the iterative approach combining PennCNV and visual evaluation resulted in precision of at least 98% (more likely 100% since the 7 exceptions were probably *de novo* deletions) and a projected sensitivity of 94%.

We consider the transmitted deletions to be, as a group, the ‘gold standard’ with respect to our level of confidence in their authenticity, because of the reciprocal validation that the trio structure afforded. On the other hand, even though the non-transmitted and *de novo* deletions did not have the benefit of this internal validation, we also have a high level of confidence in their authenticity since the reproducibility of the deletion calls from the two generations indicates that the methodology and overall approach to identifying deletions is sound. Furthermore, several of the deletions found in the SZ cohort (without the availability of parents) were validated by qPCR (below) and 100% coincidence was found between gene dosage predicted from BeadStudio data and that measured by the PCR technology.

**Validation of rare deletions**

Relative gene copy numbers were determined in the DNA samples described below by real-time quantitative PCR (qPCR) using a PRISM® 7900HT Sequence Detection System (384 wells; Applied Biosystems, Foster City, CA). The PCR reactions were carried out using the SYBR® Green PCR Master Mix (Applied Biosystems, Foster City, CA), according to the manufacturer’s instructions. PCR conditions were as follows: 10 min at 95°C, followed by 45 two-step cycles of (15 s at 95°C, 60 s at 60°C) and a final cycle of (15 s at 95°C, 15 s at 60°C, 15 s at 95°C). Primers (Table S3) were designed using Primer 3 and synthesized by Integrated DNA Technologies (IDT; Coralville, IA). Uniqueness of primers and amplicons was checked using BLAT and in-silico-PCR against the hg18 reference assembly [2]. For each primer, the final concentration was optimized over three concentrations (50 nM, 300 nM and 900 nM). The optimal concentration was that which obtained the lowest threshold cycle (Ct) and maximum ΔRn while minimizing non-specific amplification, as described in the manufacturer’s literature.

Putative rare deletions in neurexin 1 gene (NRXN1) were investigated using 5 primer pairs targeting different regions of the gene suspected to be deleted in at least one sample. One primer pair, designed in an unaffected region of the gene was included as a control. Finally, an additional primer pair was designed in the H6PD gene and use as an internal control for copy number and for normalization.

Data were normalized and Relative Copy Number determined using the ΔKCt method as described by Weksberg et al. [5]. Accordingly, the final results are expressed in ΔKCt values where values of 0 ± 0.35 indicate no probable copy number variation, -1 ± 0.35 indicate the probable loss of one copy (deletion of one allele), and 1 ± 0.35 indicate the probable gain of one copy (duplication of one allele).

**References**

1. McCarroll SA et al. (2006) [Common deletion polymorphisms in the human genome](http://www.ncbi.nlm.nih.gov/pubmed/16468122?ordinalpos=20&itool=EntrezSystem2.PEntrez.Pubmed.Pubmed_ResultsPanel.Pubmed_DefaultReportPanel.Pubmed_RVDocSum). *Nat Genet* 38:86-92.

2. Wang K et al. (2007) PennCNV: an integrated hidden Markov model designed for high-resolution copy number variation detection in whole-genome SNP genotyping data. *Genome Res* 17:1665-1674

3. Rozen S, Skaletsky H (2000) in *Bioinformatics Methods and Protocols: Methods in Molecular Biology,* eds Krawetz S, Misener S (Humana Press, Totowa, NJ), pp 365-386

4. Kent WJ (2002) BLAT - the BLAST-like alignment tool. *Genome Res.* 12:656–664.

5. Weksberg R (2005) A method for accurate detection of genomic microdeletions using real-time quantitative PCR. *BMC Genomics* 6:180.

6. Sironi M et al. (2006) A region in the dystrophin gene major hot spot harbors a cluster of deletion breakpoints and generates double-strand breaks in yeast. *FASEB J* 20:1910-1912

7. Blauw HM et al. (2008) Copy-number variation in sporadic amyotrophic lateral sclerosis: a genome-wide screen. *Lancet Neurol* 7:319-326.

**Figure Legends**

Figure S1

A sequence of 9 consecutive BeadStudio images showing the six-SNP *de novo* deletion illustrated in Fig. 2 of the main text. The offspring with the deletion as well as the parents are highlighted (yellow). A trio with apparent non-Mendelian transmission is indicated by BeadStudio as circles (mother and offspring) or X (father). Note that in both BeadStudio clusters of individual SNPs (this figure) and the Genome Viewer display of all SNPs in a given region for the one individual (Fig. 2, main text) the reduced intensity (expressed as ‘Norm R’ and ‘log R ratio’ respectively) is evident. In addition, note that in several panels of this figure at least one other sample yields a data point of reduced Norm R. For 3 consecutive SNPs beginning at rs6042809 the same sample (from a father in a different trio) yielded the values close to those for the *de novo* deletion; this was scored as a non-transmitted deletion. Each of the other points below the cluster was from a different individual, and they were considered as outliers.

Figure S2

Superimposition of logR ratios from two trios within regions each carrying a 3-SNP transmitted deletion. The LogR ratio data were exported from BeadStudio software into Tableau, normalized per sample and displayed to demonstrate the reproducibility of the reduction in Log R ratio in the transmitting parent and the child. A, Chromosome 11, mother transmitting; B, Chromosome 20, father transmitting. Insets show expanded x-axes.

Figure S3

Validation of deletions in the NRXN1 gene by qPCR. LogR ratios from Illumina’s Genome Viewer are shown in the left panel of each quadrant, with the location of the primers depicted above the gene. The respective right panels of each quadrant show histograms of ΔKCt, the copy number change from diploidy, relative to the H6PD gene, at far right of each histogram.

Figure S1


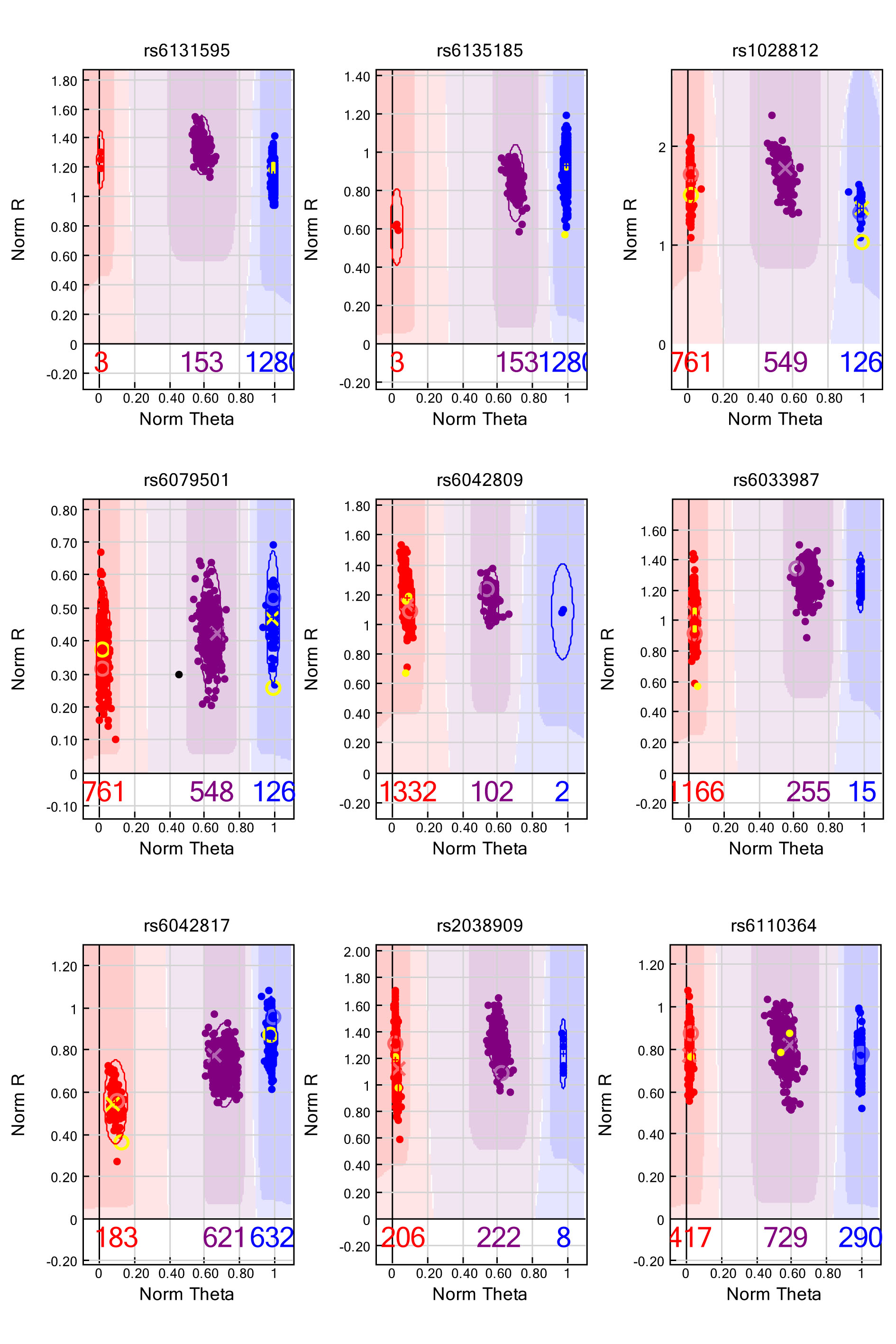


Figure S2


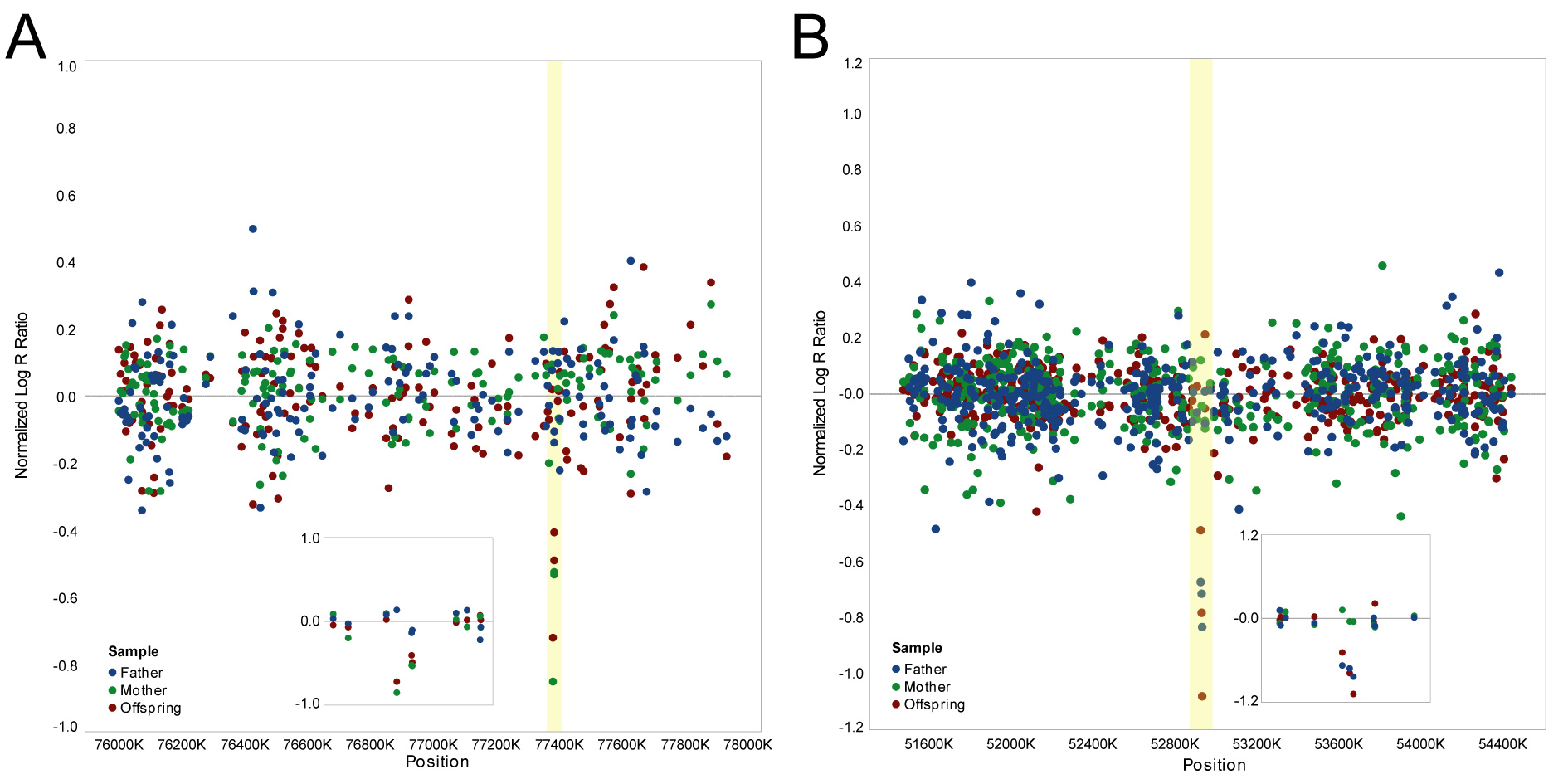


Figure S3


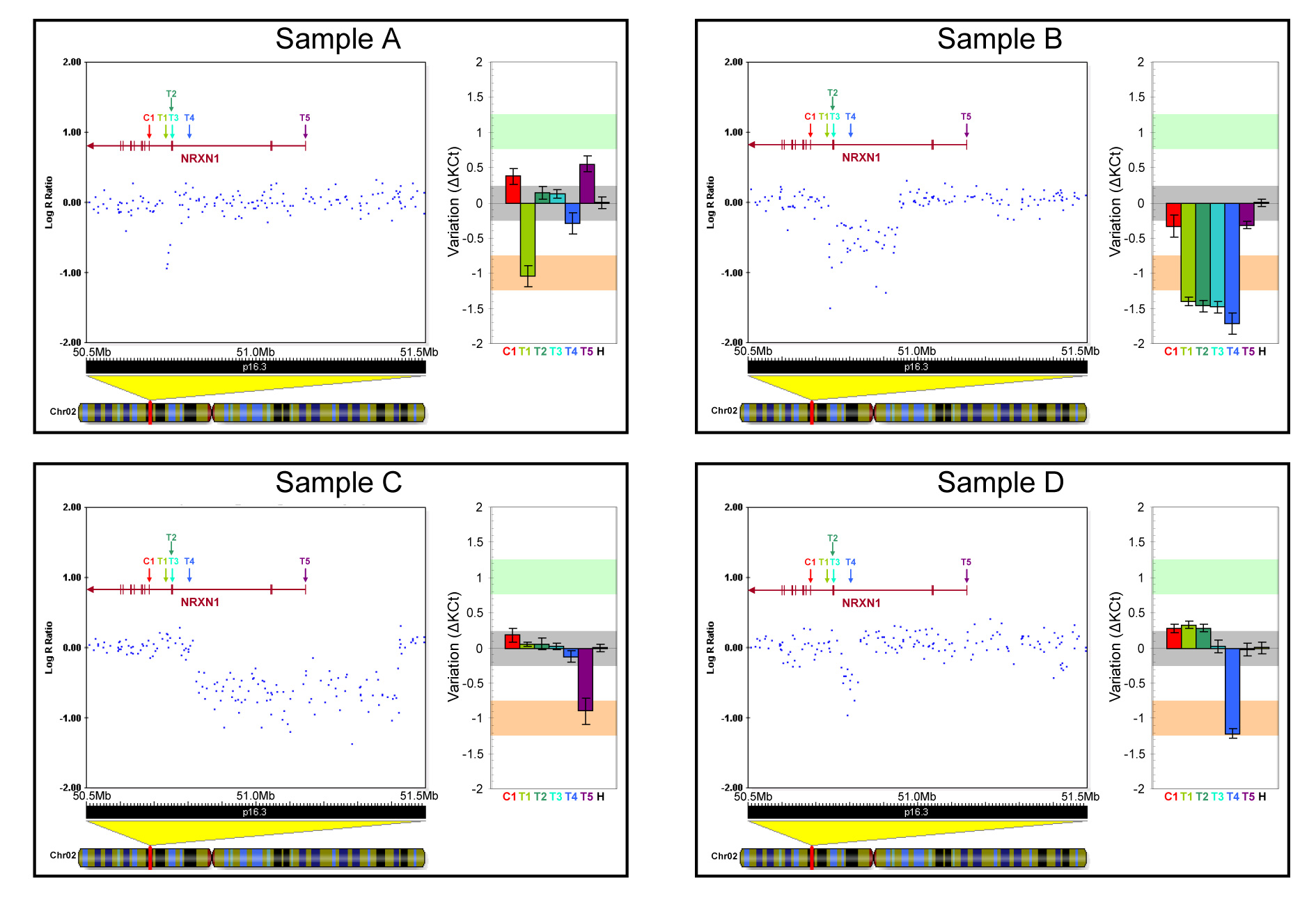


Table S1.

List of 368 independent affirmed rare deletions in offspring of the ADHD sample (440 parent-parent trios). Seven deletions were *de novo*; all others (transmitted deletions) showed perfect concordance between parent and child.

|  |  |  |  |  |  |  |  |
| --- | --- | --- | --- | --- | --- | --- | --- |
| Marker | DNA ID | Log Bayes Factor | No. of SNPs | chr | Start (Build 35) | End | Length (Mb) |
| rs875727 | 281 | 51.619 | 10 | 1 | 71.086 | 71.167 | 0.081 |
| rs4384177 | 210 | 18.94 | 4 | 1 | 73.694 | 73.719 | 0.025 |
| rs6424687 | 303 | 39.349 | 8 | 1 | 80.254 | 80.325 | 0.071 |
| rs6699405 | 337 | 27.248 | 5 | 1 | 83.971 | 83.979 | 0.008 |
| rs1721525 | 101 | 16.088 | 3 | 1 | 105.758 | 105.776 | 0.018 |
| rs157880 | 178 | 18.931 | 4 | 1 | 162.133 | 162.145 | 0.012 |
| rs507603 | 290 | 17.179 | 3 | 1 | 176.63 | 176.639 | 0.009 |
| rs2211195 | 29 | 28.301 | 8 | 1 | 188.568 | 188.636 | 0.068 |
| rs1339307 | 288 | 35.237 | 7 | 1 | 190.321 | 190.406 | 0.085 |
| rs4557953 | 53 | 42.117 | 10 | 1 | 191.445 | 191.548 | 0.103 |
| rs6540742 | 75 | 15.67 | 3 | 1 | 209.059 | 209.064 | 0.005 |
| rs12749729 | 264 | 258.232 | 59 | 1 | 218.272 | 218.637 | 0.365 |
| rs2793086 | 308 | 56.397 | 15 | 1 | 228.206 | 228.267 | 0.061 |
| rs12137417 | 230 | 69.782 | 17 | 1 | 228.393 | 228.507 | 0.114 |
| rs1033322 | 9 | 58.798 | 15 | 1 | 229.707 | 229.789 | 0.082 |
| rs780242 | 171 | 18.847 | 3 | 1 | 229.995 | 230.001 | 0.006 |
| rs10754573 | 281 | 21.269 | 4 | 1 | 233.004 | 233.01 | 0.006 |
| rs6693951 | 176 | 14.545 | 3 | 1 | 235.278 | 235.282 | 0.004 |
| rs9428429 | 73 | 16.735 | 3 | 1 | 235.417 | 235.421 | 0.004 |
| rs1915264 | 244 | 31.262 | 6 | 1 | 235.499 | 235.551 | 0.052 |
| rs6703086 | 98 | 21.106 | 4 | 1 | 235.67 | 235.682 | 0.012 |
| rs4241338 | 22 | 36.02 | 9 | 2 | 0.872 | 0.983 | 0.111 |
| rs4241338 | 128 | 13.51 | 5 | 2 | 5.837 | 5.85 | 0.013 |
| rs12470356 | 344 | 55.861 | 12 | 2 | 18.107 | 18.174 | 0.067 |
| rs1862985 | 246 | 15.304 | 3 | 2 | 31.184 | 31.19 | 0.006 |
| rs1371422 | 77 | 16.59 | 4 | 2 | 35.626 | 35.691 | 0.065 |
| rs11124448 | 118 | 14.912 | 3 | 2 | 35.684 | 35.691 | 0.007 |
| rs232579 | 4 | 76.822 | 17 | 2 | 38.296 | 38.375 | 0.079 |
| rs7599875 | 4 | 32.541 | 6 | 2 | 41.923 | 41.93 | 0.007 |
| rs17031289 | 17 | 14.47 | 4 | 2 | 43.836 | 43.843 | 0.007 |
| rs713446 | 221 | 14.99 | 9 | 2 | 44.147 | 44.496 | 0.349 |
| rs1517027 | 236 | 36.817 | 8 | 2 | 45.244 | 45.274 | 0.03 |
| rs4245815 | 161 | 16.032 | 3 | 2 | 47.732 | 47.734 | 0.002 |
| rs11125149 | 239 | 18.496 | 4 | 2 | 47.791 | 47.802 | 0.011 |
| rs6755968 | 186 | 33.84 | 7 | 2 | 48.355 | 48.405 | 0.05 |
| rs2293276 | 314 | 16.698 | 3 | 2 | 48.732 | 48.739 | 0.007 |
| rs3901491 | 153 | 33.285 | 6 | 2 | 50.888 | 50.914 | 0.026 |
| rs9309199 | 95 | 170.26 | 37 | 2 | 50.925 | 51.11 | 0.186 |
| rs6740594 | 147 | 16.253 | 3 | 2 | 56.814 | 56.84 | 0.026 |
| rs10179966 | 152 | 16.092 | 5 | 2 | 97.267 | 97.625 | 0.358 |
| rs1922296 | 41 | 16.331 | 3 | 2 | 102.281 | 102.286 | 0.005 |
| rs13009482 | 141 | 13.39 | 3 | 2 | 102.981 | 102.983 | 0.002 |
| rs272128 | 131 | 150.436 | 38 | 2 | 131.321 | 131.911 | 0.59 |
| rs12990583 | 262 | 116.811 | 27 | 2 | 137.763 | 138.048 | 0.285 |
| rs17752647 | 169 | 116.833 | 24 | 2 | 139.643 | 139.838 | 0.195 |
| rs1511199 | 174 | 16.583 | 4 | 2 | 167.73 | 167.741 | 0.011 |
| rs1993269 | 311 | 16.497 | 3 | 2 | 184.407 | 184.43 | 0.023 |
| rs1521658 | 266 | 82.635 | 16 | 2 | 212.836 | 212.915 | 0.079 |
| rs1898710 | 106 | 14.656 | 3 | 2 | 216.311 | 216.316 | 0.005 |
| rs7603146 | 265 | 15.578 | 3 | 2 | 234.514 | 234.519 | 0.005 |
| rs9681213 | 92 | 1347.681 | 286 | 3 | 0.042 | 1.623 | 1.581 |
| rs9837987 | 212 | 48.23 | 10 | 3 | 0.574 | 0.686 | 0.112 |
| rs1110797 | 192 | 79.043 | 16 | 3 | 4.007 | 4.086 | 0.079 |
| rs7638636 | 351 | 15.041 | 3 | 3 | 6.735 | 6.741 | 0.006 |
| rs2017903 | 349 | 17.662 | 3 | 3 | 10.801 | 10.809 | 0.008 |
| rs1979789 | 266 | 46.958 | 10 | 3 | 42.152 | 42.209 | 0.057 |
| rs9881520 | 174 | 374.993 | 98 | 3 | 61.601 | 62.062 | 0.461 |
| rs9863159 | 187 | 65.127 | 14 | 3 | 61.631 | 61.695 | 0.064 |
| rs6781900 | 342 | 16.751 | 3 | 3 | 74.047 | 74.051 | 0.004 |
| rs1580295 | 339 | 19.576 | 7 | 3 | 82.798 | 82.849 | 0.051 |
| rs11710892 | 279 | 22.782 | 7 | 3 | 83.7 | 83.717 | 0.017 |
| rs11713152 | 299 | 14.822 | 4 | 3 | 84.936 | 84.957 | 0.021 |
| rs2117152 | 256 | 16.978 | 3 | 3 | 85.487 | 85.497 | 0.01 |
| rs6437568 | 99 | 12.765 | 5 | 3 | 106.371 | 106.391 | 0.02 |
| rs340063 | 294 | 399.062 | 91 | 3 | 107.199 | 107.985 | 0.786 |
| rs7612247 | 182 | 12.79 | 3 | 3 | 110.732 | 110.739 | 0.007 |
| rs6783347 | 31 | 33.573 | 6 | 3 | 111.718 | 111.797 | 0.079 |
| rs2077807 | 184 | 31.178 | 7 | 3 | 146.708 | 146.736 | 0.028 |
| rs1400612 | 297 | 17.788 | 3 | 3 | 153.833 | 153.837 | 0.004 |
| rs6665 | 114 | 314.197 | 64 | 3 | 156.065 | 156.663 | 0.598 |
| rs9876553 | 116 | 19.098 | 4 | 3 | 162.47 | 162.527 | 0.057 |
| rs75239 | 37 | 39.097 | 10 | 3 | 163.92 | 163.994 | 0.074 |
| rs291939 | 131 | 41.375 | 8 | 3 | 175.236 | 175.301 | 0.065 |
| rs4571187 | 19 | 87.789 | 30 | 3 | 193.536 | 193.758 | 0.222 |
| rs10516174 | 272 | 139.92 | 32 | 4 | 5.204 | 5.435 | 0.231 |
| rs7697255 | 84 | 20.161 | 5 | 4 | 8.017 | 8.023 | 0.006 |
| rs4328901 | 198 | 46.31 | 11 | 4 | 25.405 | 25.442 | 0.037 |
| rs6531383 | 134 | 27.824 | 5 | 4 | 35.499 | 35.527 | 0.028 |
| rs2588681 | 355 | 15.972 | 3 | 4 | 66.778 | 66.799 | 0.021 |
| rs3775866 | 240 | 15.323 | 3 | 4 | 68.2729 | 68.273 | 1.00E-04 |
| rs1460013 | 361 | 26.243 | 9 | 4 | 75.623 | 75.681 | 0.058 |
| rs7438530 | 296 | 27.71 | 5 | 4 | 79.487 | 79.507 | 0.02 |
| rs7688441 | 42 | 15.538 | 4 | 4 | 84.384 | 84.415 | 0.031 |
| rs10516853 | 298 | 31.228 | 7 | 4 | 91.224 | 91.31 | 0.086 |
| rs4692966 | 207 | 21.346 | 5 | 4 | 93.373 | 93.419 | 0.046 |
| rs1479324 | 366 | 67.54 | 14 | 4 | 93.936 | 94.064 | 0.128 |
| rs2063543 | 316 | 185.059 | 41 | 4 | 104.717 | 105.202 | 0.485 |
| rs9307620 | 195 | 25.641 | 7 | 4 | 131.321 | 131.374 | 0.053 |
| rs976259 | 134 | 25.514 | 5 | 4 | 134.511 | 134.581 | 0.07 |
| rs1390009 | 114 | 108.56 | 23 | 4 | 135.29 | 135.534 | 0.244 |
| rs6844670 | 276 | 23.008 | 4 | 4 | 145.382 | 145.391 | 0.009 |
| rs466478 | 319 | 57.19 | 11 | 4 | 163.258 | 163.331 | 0.073 |
| rs333736 | 193 | 97.648 | 22 | 4 | 172.638 | 172.891 | 0.253 |
| rs1565469 | 299 | 13.731 | 4 | 4 | 172.928 | 172.962 | 0.034 |
| rs3108252 | 183 | 50.367 | 11 | 4 | 186.524 | 186.611 | 0.087 |
| rs4863164 | 62 | 17.501 | 3 | 4 | 189.47 | 189.477 | 0.007 |
| rs1995038 | 325 | 97.518 | 28 | 5 | 7.026 | 7.238 | 0.212 |
| rs10057809 | 16 | 41.493 | 10 | 5 | 13.535 | 13.584 | 0.049 |
| rs964751 | 77 | 51.094 | 16 | 5 | 17.434 | 17.499 | 0.065 |
| rs7709236 | 259 | 21.142 | 4 | 5 | 21.439 | 21.476 | 0.037 |
| rs301904 | 249 | 32.722 | 6 | 5 | 37.147 | 37.242 | 0.095 |
| rs2052477 | 319 | 13.72 | 3 | 5 | 77.473 | 77.509 | 0.036 |
| rs655680 | 336 | 22.127 | 6 | 5 | 81.253 | 81.281 | 0.028 |
| rs4920821 | 232 | 155.928 | 30 | 5 | 84.474 | 84.666 | 0.192 |
| rs2948768 | 275 | 33.995 | 6 | 5 | 98.728 | 98.807 | 0.079 |
| rs13190093 | 347 | 24.234 | 6 | 5 | 99.361 | 99.415 | 0.054 |
| rs1504530 | 228 | 82.517 | 19 | 5 | 99.562 | 99.821 | 0.259 |
| rs354652 | 137 | 34.729 | 6 | 5 | 101.165 | 101.231 | 0.066 |
| rs322854 | 18 | 44.373 | 15 | 5 | 104.254 | 104.34 | 0.086 |
| rs319915 | 36 | 26.787 | 5 | 5 | 104.3 | 104.316 | 0.016 |
| rs4246006 | 95 | 39.005 | 8 | 5 | 109.628 | 109.664 | 0.036 |
| rs7442670 | 356 | 16.094 | 3 | 5 | 114.387 | 114.396 | 0.009 |
| rs7724716 | 107 | 18.517 | 4 | 5 | 130.158 | 130.169 | 0.011 |
| rs2740583 | 281 | 25.308 | 5 | 5 | 140.54 | 140.571 | 0.031 |
| rs6885316 | 109 | 16.315 | 3 | 5 | 152.258 | 152.269 | 0.011 |
| rs4379221 | 212 | 1503.667 | 272 | 5 | 164.216 | 166.447 | 2.231 |
| rs347434 | 227 | 20.277 | 5 | 5 | 166.032 | 166.059 | 0.027 |
| rs9392279 | 126 | 19.686 | 4 | 6 | 1.295 | 1.311 | 0.016 |
| rs4959410 | 44 | 22.476 | 4 | 6 | 6.748 | 6.753 | 0.005 |
| rs645297 | 90 | NA | 12 | 6 | 10.578 | 10.637 | 0.059 |
| rs449242 | 136 | 17.582 | 5 | 6 | 33.605 | 33.634 | 0.029 |
| rs2817056 | 23 | 31.509 | 6 | 6 | 35.842 | 35.858 | 0.016 |
| rs9294730 | 61 | 31.969 | 9 | 6 | 67.806 | 67.914 | 0.108 |
| rs3793027 | 71 | 346.004 | 67 | 6 | 70.728 | 71.105 | 0.377 |
| rs2307389 | 301 | 21.455 | 4 | 6 | 88.376 | 88.395 | 0.019 |
| rs382078 | 242 | 52.387 | 11 | 6 | 95.284 | 95.366 | 0.082 |
| rs794669 | 238 | NA | 5 | 6 | 95.561 | 95.614 | 0.053 |
| rs794669 | 338 | 14.646 | 3 | 6 | 95.561 | 95.57 | 0.009 |
| rs1901708 | 233 | 19.314 | 4 | 6 | 105.026 | 105.037 | 0.011 |
| rs6903177 | 68 | 15.82 | 4 | 6 | 118.802 | 118.833 | 0.031 |
| rs8192627 | 145 | 28.151 | 6 | 6 | 132.917 | 132.931 | 0.014 |
| rs1856756 | 201 | 66.047 | 15 | 6 | 140.772 | 141.089 | 0.317 |
| rs2502637 | 293 | 28.776 | 6 | 6 | 144.651 | 144.672 | 0.021 |
| rs1954925 | 346 | 24.658 | 6 | 6 | 162.498 | 162.542 | 0.044 |
| rs952901 | 190 | 125.61 | 22 | 6 | 162.568 | 162.71 | 0.142 |
| rs951196 | 207 | 18.33 | 5 | 6 | 162.632 | 162.68 | 0.048 |
| rs7751716 | 206 | 106.77 | 21 | 6 | 162.674 | 162.779 | 0.105 |
| rs7806592 | 3 | 27.6 | 12 | 7 | 0.162 | 0.219 | 0.057 |
| rs4343997 | 221 | 15.059 | 6 | 7 | 3.118 | 3.182 | 0.064 |
| rs4400288 | 44 | 13.607 | 3 | 7 | 4.93 | 4.971 | 0.041 |
| rs4724739 | 314 | 12.974 | 3 | 7 | 5.624 | 5.664 | 0.04 |
| rs11765838 | 70 | 43.481 | 9 | 7 | 11.09 | 11.132 | 0.042 |
| rs7785249 | 291 | 107.345 | 20 | 7 | 14.087 | 14.221 | 0.134 |
| rs1111544 | 292 | 16.563 | 5 | 7 | 17.188 | 17.227 | 0.039 |
| rs1989886 | 35 | 36.74 | 7 | 7 | 20.356 | 20.378 | 0.022 |
| rs6974500 | 229 | 18.754 | 4 | 7 | 23.518 | 23.537 | 0.019 |
| rs6974169 | 146 | 54.66 | 16 | 7 | 62.981 | 63.227 | 0.246 |
| rs17468652 | 49 | 49.841 | 12 | 7 | 75.781 | 76.155 | 0.374 |
| rs1024507 | 92 | 64.585 | 13 | 7 | 88.216 | 88.302 | 0.086 |
| rs2215443 | 111 | 44.788 | 9 | 7 | 89.17 | 89.215 | 0.045 |
| rs177667 | 304 | 30.051 | 5 | 7 | 89.745 | 89.763 | 0.018 |
| rs1965519 | 165 | 17.705 | 4 | 7 | 100.954 | 100.975 | 0.021 |
| rs6976282 | 350 | 14.269 | 4 | 7 | 101.619 | 101.643 | 0.024 |
| rs1204564 | 362 | 15.693 | 3 | 7 | 126.184 | 126.189 | 0.005 |
| rs4728329 | 69 | 13.271 | 3 | 7 | 133.683 | 133.711 | 0.028 |
| rs2471312 | 28 | 13.591 | 3 | 7 | 139.422 | 139.433 | 0.011 |
| rs6947359 | 127 | 20.02 | 6 | 7 | 141.776 | 141.802 | 0.026 |
| rs11762140 | 155 | 66.269 | 15 | 7 | 149.699 | 149.835 | 0.136 |
| rs1986588 | 53 | 23.253 | 6 | 7 | 157.515 | 157.56 | 0.045 |
| rs17752600 | 254 | 86.554 | 19 | 8 | 2.099 | 2.155 | 0.056 |
| rs2623638 | 13 | 24.054 | 6 | 8 | 3.616 | 3.633 | 0.017 |
| rs1217680 | 87 | 31.739 | 8 | 8 | 4.302 | 4.323 | 0.021 |
| rs10503274 | 94 | 20.491 | 4 | 8 | 4.593 | 4.594 | 0.0008 |
| rs4242499 | 159 | 31.163 | 6 | 8 | 4.867 | 4.897 | 0.03 |
| rs1588676 | 142 | 61.557 | 17 | 8 | 5.672 | 5.768 | 0.096 |
| rs7840430 | 71 | 13.865 | 3 | 8 | 6.152 | 6.159 | 0.007 |
| rs736227 | 63 | 14.537 | 3 | 8 | 6.781 | 6.783 | 0.002 |
| rs712255 | 11 | NA | 6 | 8 | 8.157 | 8.193 | 0.036 |
| rs6993771 | 311 | 14.164 | 3 | 8 | 15.23 | 15.241 | 0.011 |
| rs1546149 | 86 | 17.02 | 3 | 8 | 15.377 | 15.386 | 0.009 |
| rs12547525 | 319 | 23.192 | 4 | 8 | 15.465 | 15.472 | 0.008 |
| rs1864236 | 34 | 29.387 | 6 | 8 | 15.533 | 15.556 | 0.023 |
| rs4461937 | 190 | 66.096 | 14 | 8 | 16.461 | 16.569 | 0.108 |
| rs1357558 | 328 | 25.161 | 6 | 8 | 16.857 | 16.87 | 0.013 |
| rs2035681 | 207 | 17.039 | 5 | 8 | 18.493 | 18.497 | 0.004 |
| rs2222361 | 335 | 13.99 | 3 | 8 | 80.208 | 80.223 | 0.015 |
| rs1524838 | 332 | 96.654 | 19 | 8 | 88.062 | 88.187 | 0.125 |
| rs318249 | 302 | 12.793 | 3 | 8 | 90.275 | 90.28 | 0.005 |
| rs11783877 | 138 | 15.551 | 3 | 8 | 98.263 | 98.274 | 0.011 |
| rs687279 | 2 | 25.328 | 5 | 8 | 128.409 | 128.426 | 0.017 |
| rs2181515 | 223 | 44.422 | 10 | 9 | 1.65 | 1.681 | 0.031 |
| rs970151 | 111 | 48.136 | 10 | 9 | 3.11 | 3.144 | 0.034 |
| rs6476866 | 233 | 16.811 | 3 | 9 | 4.449 | 4.456 | 0.007 |
| rs1571221 | 67 | 21.277 | 5 | 9 | 4.926 | 4.933 | 0.007 |
| rs2054314 | 21 | 13.362 | 4 | 9 | 6.281 | 6.312 | 0.031 |
| rs7870809 | 1 | 22.408 | 4 | 9 | 6.558 | 6.576 | 0.018 |
| rs2918182 | 365 | 16.451 | 5 | 9 | 6.654 | 6.659 | 0.005 |
| rs324498 | 205 | 15.1 | 3 | 9 | 9.05 | 9.058 | 0.008 |
| rs10959084 | 258 | 29.214 | 7 | 9 | 10.372 | 10.437 | 0.065 |
| rs10123266 | 360 | 27.846 | 6 | 9 | 11.189 | 11.265 | 0.076 |
| rs1580540 | 308 | 154.106 | 37 | 9 | 11.295 | 11.674 | 0.379 |
| rs10960174 | 226 | 53.326 | 12 | 9 | 11.744 | 11.838 | 0.094 |
| rs10960278 | 56 | 112.773 | 22 | 9 | 11.81 | 12.038 | 0.228 |
| rs7039047 | 104 | 19.617 | 4 | 9 | 11.936 | 11.95 | 0.014 |
| rs10809666 | 214 | 100.94 | 20 | 9 | 11.993 | 12.114 | 0.121 |
| rs791777 | 274 | 88.546 | 17 | 9 | 12.14 | 12.23 | 0.09 |
| rs791688 | 187 | 27.466 | 5 | 9 | 12.517 | 12.523 | 0.006 |
| rs3824388 | 142 | 84.283 | 19 | 9 | 17.615 | 17.649 | 0.034 |
| rs7862129 | 334 | 27.481 | 5 | 9 | 18.28 | 18.286 | 0.006 |
| rs7027485 | 101 | 51.955 | 11 | 9 | 19.235 | 19.333 | 0.098 |
| rs10967655 | 142 | 22.103 | 6 | 9 | 26.994 | 27.037 | 0.043 |
| rs10491619 | 304 | 129.865 | 26 | 9 | 28.674 | 28.795 | 0.121 |
| rs1418344 | 341 | 60.094 | 18 | 9 | 29.075 | 29.237 | 0.162 |
| rs3853275 | 272 | 40.887 | 8 | 9 | 30.434 | 30.548 | 0.114 |
| rs3853275 | 308 | 43.069 | 10 | 9 | 30.434 | 30.625 | 0.191 |
| rs1857553 | 161 | 19.529 | 4 | 9 | 30.536 | 30.625 | 0.089 |
| rs1335145 | 196 | 453.051 | 90 | 9 | 31.1 | 32.111 | 1.011 |
| rs12001157 | 24 | 23.321 | 4 | 9 | 69.33 | 69.349 | 0.019 |
| rs4556160 | 364 | 16.239 | 3 | 9 | 79.262 | 79.263 | 0.001 |
| rs2472519 | 55 | 60.009 | 12 | 9 | 104.796 | 104.834 | 0.038 |
| rs7022165 | 185 | 19.556 | 4 | 9 | 112.932 | 112.947 | 0.015 |
| rs868693 | 80 | 15.925 | 3 | 9 | 123.13 | 123.139 | 0.009 |
| rs7852711 | 269 | 21.653 | 4 | 9 | 131.263 | 131.269 | 0.006 |
| rs7082636 | 120 | 15.577 | 3 | 10 | 4.812 | 4.819 | 0.007 |
| rs11253059 | 39 | 15.386 | 3 | 10 | 5.265 | 5.276 | 0.011 |
| rs1756754 | 261 | 17.453 | 4 | 10 | 30.269 | 30.287 | 0.018 |
| rs7895874 | 83 | 179.079 | 42 | 10 | 56.548 | 56.784 | 0.236 |
| rs11005017 | 328 | 71.669 | 17 | 10 | 57.207 | 57.349 | 0.142 |
| rs10509267 | 159 | 59.716 | 14 | 10 | 67.948 | 68.044 | 0.096 |
| rs2659998 | 165 | 94.671 | 24 | 10 | 67.963 | 68.118 | 0.155 |
| rs6480182 | 306 | 67.804 | 8 | 10 | 68.034 | 68.069 | 0.035 |
| rs780668 | 327 | 34.796 | 11 | 10 | 72.768 | 72.792 | 0.024 |
| rs7919006 | 173 | 22.541 | 8 | 10 | 76.479 | 76.513 | 0.034 |
| rs1739765 | 288 | 16.079 | 3 | 10 | 83.94 | 83.944 | 0.004 |
| rs1772810 | 97 | 14.238 | 3 | 10 | 119.147 | 119.161 | 0.014 |
| rs10788106 | 250 | 22.52 | 4 | 10 | 122.382 | 122.385 | 0.003 |
| rs366663 | 15 | 19.588 | 5 | 10 | 132.073 | 132.086 | 0.013 |
| rs2265900 | 202 | 85.327 | 17 | 10 | 135.155 | 135.266 | 0.111 |
| rs4351775 | 317 | 21.449 | 5 | 10 | 135.291 | 135.323 | 0.032 |
| rs7937618 | 100 | 16.568 | 4 | 11 | 1.604 | 1.61 | 0.006 |
| rs1512829 | 284 | 14.101 | 3 | 11 | 9.908 | 9.93 | 0.022 |
| rs12282103 | 82 | 35.054 | 8 | 11 | 13.759 | 13.82 | 0.061 |
| rs7924783 | 352 | 16.327 | 3 | 11 | 16.49 | 16.532 | 0.042 |
| rs7105978 | 59 | 49.379 | 11 | 11 | 21.357 | 21.386 | 0.029 |
| rs2128958 | 26 | 21.158 | 5 | 11 | 28.121 | 28.217 | 0.096 |
| rs2289989 | 151 | 24.791 | 7 | 11 | 36.247 | 36.278 | 0.031 |
| rs1039205 | 219 | 31.939 | 14 | 11 | 36.668 | 36.782 | 0.114 |
| rs7125435 | 120 | 36.594 | 6 | 11 | 40.996 | 41.02 | 0.024 |
| rs17093 | 279 | 17.571 | 4 | 11 | 44.987 | 45.007 | 0.02 |
| rs668095 | 353 | 56.2 | 12 | 11 | 59.757 | 59.818 | 0.061 |
| rs4944158 | 307 | 18.95 | 4 | 11 | 76.704 | 76.776 | 0.072 |
| rs3824865 | 165 | 14.108 | 3 | 11 | 77.405 | 77.409 | 0.004 |
| rs2375030 | 103 | 16.516 | 4 | 11 | 86.855 | 86.865 | 0.01 |
| rs6588953 | 84 | 26.107 | 6 | 11 | 106.62 | 106.663 | 0.043 |
| rs2298831 | 331 | 17.452 | 3 | 11 | 117.574 | 117.58 | 0.006 |
| rs1944816 | 257 | 17.319 | 4 | 11 | 126.557 | 126.563 | 0.006 |
| rs528420 | 133 | 17.214 | 3 | 11 | 134.268 | 134.279 | 0.011 |
| rs10772683 | 214 | 16.447 | 3 | 12 | 13.527 | 13.533 | 0.006 |
| rs4764371 | 52 | 38.257 | 11 | 12 | 18.118 | 18.207 | 0.089 |
| rs1875059 | 285 | 13.279 | 3 | 12 | 32.594 | 32.598 | 0.004 |
| rs1495776 | 89 | 15.989 | 3 | 12 | 40.344 | 40.344 | 0.0003 |
| rs11175055 | 122 | 23.273 | 6 | 12 | 62.269 | 62.415 | 0.146 |
| rs17827216 | 277 | 25.915 | 5 | 12 | 65.476 | 65.494 | 0.018 |
| rs10047619 | 19 | 13.819 | 5 | 12 | 81.671 | 81.723 | 0.052 |
| rs1298729 | 157 | 20.527 | 5 | 12 | 98.485 | 98.494 | 0.009 |
| rs11110069 | 218 | 32.851 | 7 | 12 | 98.744 | 98.795 | 0.051 |
| rs4760566 | 176 | 38.489 | 12 | 12 | 127.726 | 127.793 | 0.067 |
| rs3874198 | 317 | 16.202 | 4 | 13 | 18.133 | 18.21 | 0.077 |
| rs745125 | 357 | 15.834 | 3 | 13 | 33.795 | 33.807 | 0.012 |
| rs2050590 | 237 | 100.876 | 20 | 13 | 37.501 | 37.68 | 0.179 |
| rs7333314 | 285 | 16.256 | 3 | 13 | 44.234 | 44.24 | 0.006 |
| rs474398 | 324 | 43.575 | 8 | 13 | 62.625 | 62.725 | 0.1 |
| rs1323922 | 285 | 154.914 | 30 | 13 | 66.201 | 66.47 | 0.269 |
| rs9540966 | 43 | 25.198 | 6 | 13 | 66.474 | 66.502 | 0.028 |
| rs495593 | 179 | 191.602 | 26 | 13 | 71.818 | 71.945 | 0.127 |
| rs7987156 | 117 | 19.038 | 4 | 13 | 83.149 | 83.161 | 0.012 |
| rs7325634 | 12 | 19.524 | 6 | 13 | 83.2135 | 83.258 | 0.045 |
| rs928602 | 65 | 55.116 | 10 | 13 | 83.375 | 83.474 | 0.099 |
| rs1928920 | 363 | 17.253 | 3 | 13 | 83.668 | 83.705 | 0.037 |
| rs2497421 | 37 | 43.92 | 8 | 13 | 84.772 | 84.884 | 0.112 |
| rs970028 | 58 | 40.553 | 8 | 13 | 87.375 | 87.421 | 0.046 |
| rs970028 | 319 | 19.606 | 4 | 13 | 94.955 | 94.968 | 0.013 |
| rs12583114 | 165 | 20.328 | 5 | 13 | 97.562 | 97.581 | 0.019 |
| rs1329515 | 87 | 21.029 | 5 | 13 | 102.495 | 102.503 | 0.008 |
| rs8003288 | 157 | 50.889 | 12 | 14 | 20.195 | 20.247 | 0.052 |
| rs7153987 | 163 | 70.121 | 14 | 14 | 26.223 | 26.314 | 0.091 |
| rs4906484 | 223 | 49.01 | 9 | 14 | 43.146 | 43.236 | 0.09 |
| rs1958249 | 260 | 269.385 | 56 | 14 | 43.363 | 43.854 | 0.491 |
| rs10151195 | 235 | 31.407 | 6 | 14 | 46.721 | 46.748 | 0.027 |
| rs1958643 | 278 | 19.614 | 3 | 14 | 53.39 | 53.391 | 0.001 |
| rs956390 | 311 | 47.978 | 11 | 14 | 57.075 | 57.116 | 0.041 |
| rs4899366 | 113 | 18.712 | 4 | 14 | 70.22 | 70.232 | 0.012 |
| rs1190906 | 199 | 35.445 | 8 | 14 | 100.169 | 100.19 | 0.021 |
| rs2073670 | 148 | 21.47 | 5 | 14 | 106.185 | 106.218 | 0.033 |
| rs1110961 | 46 | NA | 3 | 15 | 21.921 | 22.036 | 0.115 |
| rs1110961 | 270 | NA | 5 | 15 | 21.921 | 22.218 | 0.297 |
| rs7497236 | 359 | 15.856 | 3 | 15 | 22.2 | 22.336 | 0.136 |
| rs1080476 | 149 | 26.477 | 6 | 15 | 48.068 | 48.095 | 0.027 |
| rs1021744 | 10 | 67.617 | 12 | 15 | 51.501 | 51.585 | 0.084 |
| rs10518725 | 64 | 133.615 | 30 | 15 | 51.603 | 51.809 | 0.206 |
| rs11629835 | 105 | 32.986 | 9 | 15 | 75.575 | 75.639 | 0.064 |
| rs11636187 | 164 | 12.753 | 3 | 15 | 79.472 | 79.499 | 0.027 |
| rs4980345 | 155 | 35.275 | 8 | 15 | 83.263 | 83.286 | 0.023 |
| rs12910436 | 262 | 18.188 | 4 | 15 | 86.71 | 86.729 | 0.019 |
| rs10520737 | 109 | 56.15 | 12 | 15 | 91.943 | 91.963 | 0.02 |
| rs11637458 | 245 | 15.433 | 3 | 15 | 93.284 | 93.288 | 0.004 |
| rs8039009 | 51 | 32.225 | 7 | 15 | 97.652 | 97.712 | 0.06 |
| rs1807833 | 66 | 17.762 | 3 | 15 | 99.524 | 99.532 | 0.008 |
| rs9788926 | 354 | 15.173 | 4 | 16 | 1.748 | 1.766 | 0.018 |
| rs16968710 | 246 | 48.848 | 10 | 16 | 17.295 | 17.353 | 0.058 |
| rs215901 | 253 | 62.484 | 12 | 16 | 21.516 | 21.648 | 0.132 |
| rs2113143 | 202 | 17.373 | 6 | 16 | 22.132 | 22.163 | 0.031 |
| rs9923017 | 200 | 27.767 | 5 | 16 | 25.864 | 25.873 | 0.009 |
| rs4788084 | 32 | 83.684 | 32 | 16 | 28.447 | 29.223 | 0.776 |
| rs8049439 | 74 | 56.883 | 14 | 16 | 28.745 | 28.95 | 0.205 |
| rs212165 | 193 | 49.499 | 12 | 16 | 70.99 | 71.314 | 0.324 |
| rs1978489 | 342 | 45.87 | 10 | 16 | 72.019 | 72.066 | 0.047 |
| rs8047051 | 250 | 26.619 | 5 | 16 | 75.28 | 75.402 | 0.122 |
| rs2707608 | 347 | 23.627 | 4 | 16 | 76.608 | 76.623 | 0.015 |
| rs4435267 | 179 | 103.637 | 25 | 16 | 78.337 | 78.468 | 0.131 |
| rs6564708 | 50 | 48.067 | 10 | 16 | 78.401 | 78.46 | 0.059 |
| rs7206532 | 252 | 15.679 | 3 | 16 | 79.048 | 79.057 | 0.009 |
| rs390112 | 4 | 44.405 | 12 | 16 | 82.797 | 82.857 | 0.06 |
| rs919422 | 326 | 99.867 | 26 | 16 | 83.05 | 83.133 | 0.083 |
| rs4600482 | 246 | 35.641 | 7 | 16 | 84.831 | 84.859 | 0.028 |
| rs222741 | 75 | 22.427 | 8 | 17 | 3.456 | 3.499 | 0.043 |
| rs2469842 | 8 | 19.128 | 3 | 17 | 18.8 | 18.806 | 0.006 |
| rs1878958 | 40 | 14.707 | 4 | 17 | 21.315 | 21.431 | 0.116 |
| rs1486748 | 59 | 41.948 | 9 | 17 | 47.764 | 47.873 | 0.109 |
| rs2452232 | 14 | 18.965 | 3 | 17 | 48.069 | 48.084 | 0.015 |
| rs9913633 | 130 | 47.376 | 11 | 17 | 48.816 | 48.876 | 0.06 |
| rs1457364 | 270 | 17.283 | 3 | 17 | 49.42 | 49.426 | 0.006 |
| rs8072471 | 7 | 17.289 | 3 | 17 | 62.798 | 62.806 | 0.008 |
| rs4798592 | 154 | 16.785 | 3 | 18 | 7.738 | 7.742 | 0.004 |
| rs1943108 | 29 | 15.471 | 3 | 18 | 48.679 | 48.681 | 0.002 |
| rs1539985 | 328 | 34.995 | 8 | 18 | 56.252 | 56.272 | 0.02 |
| rs2715290 | 220 | 23.118 | 5 | 18 | 61.884 | 61.907 | 0.023 |
| rs4806887 | 57 | 15.363 | 3 | 19 | 2.853 | 2.872 | 0.019 |
| rs385839 | 119 | 13.517 | 3 | 19 | 5.473 | 5.481 | 0.008 |
| rs11880217 | 266 | 56.199 | 11 | 19 | 12.28 | 12.449 | 0.169 |
| rs346155 | 139 | 16.65 | 4 | 19 | 13.746 | 13.755 | 0.009 |
| rs11666718 | 242 | 88.24 | 25 | 19 | 19.945 | 20.528 | 0.583 |
| rs11671929 | 123 | 21.285 | 5 | 19 | 20.223 | 20.332 | 0.109 |
| rs2043315 | 204 | 31.929 | 7 | 19 | 22.045 | 22.156 | 0.111 |
| rs501186 | 358 | 18.7 | 3 | 19 | 39.237 | 39.243 | 0.006 |
| rs10418296 | 231 | 28.569 | 6 | 19 | 58.594 | 58.66 | 0.066 |
| rs873733 | 125 | 17.125 | 3 | 19 | 61.437 | 61.444 | 0.007 |
| rs4801699 | 128 | 102.701 | 28 | 19 | 61.477 | 61.692 | 0.215 |
| rs4801433 | 216 | 13.02 | 3 | 19 | 62.338 | 62.342 | 0.004 |
| rs6041737 | 339 | 17.019 | 4 | 20 | 12.89 | 12.895 | 0.005 |
| rs6110271 | 26 | 26.871 | 5 | 20 | 14.323 | 14.344 | 0.021 |
| rs6135185 | 46 | 31.711 | 6 | 20 | 14.507 | 14.538 | 0.031 |
| rs4813165 | 124 | 182.515 | 43 | 20 | 14.655 | 14.989 | 0.334 |
| rs204104 | 25 | NA | 9 | 20 | 14.692 | 14.755 | 0.064 |
| rs204104 | 294 | 174.828 | 38 | 20 | 14.692 | 14.979 | 0.287 |
| rs365516 | 170 | 29.984 | 6 | 20 | 14.786 | 14.815 | 0.029 |
| rs6110458 | 181 | 81.942 | 18 | 20 | 14.811 | 14.914 | 0.103 |
| rs6135296 | 241 | 86.399 | 15 | 20 | 14.826 | 14.956 | 0.13 |
| rs6079602 | 46 | 59.859 | 13 | 20 | 14.832 | 14.885 | 0.053 |
| rs11086760 | 216 | 15.738 | 3 | 20 | 37.79 | 37.817 | 0.027 |
| rs230166 | 366 | 53.073 | 10 | 20 | 40.569 | 40.623 | 0.054 |
| rs6102926 | 33 | NA | 10 | 20 | 40.662 | 40.718 | 0.056 |
| rs6102926 | 345 | 22.207 | 5 | 20 | 40.662 | 40.677 | 0.015 |
| rs6102967 | 196 | 14.407 | 5 | 20 | 40.703 | 40.724 | 0.021 |
| rs6067999 | 77 | 17.423 | 6 | 20 | 49.939 | 49.966 | 0.027 |
| rs2041278 | 91 | 16.24 | 5 | 20 | 51.702 | 51.731 | 0.029 |
| rs283273 | 172 | 14.997 | 3 | 20 | 52.967 | 52.974 | 0.007 |
| rs6027060 | 368 | 16.56 | 4 | 20 | 57.736 | 57.742 | 0.006 |
| rs2826377 | 189 | 443.668 | 83 | 21 | 20.84 | 21.278 | 0.438 |
| rs2827712 | 210 | 41.852 | 8 | 21 | 23.1 | 23.131 | 0.031 |
| rs2829916 | 81 | 52.746 | 10 | 21 | 26.117 | 26.164 | 0.047 |
| rs2243552 | 333 | 40.081 | 8 | 21 | 29.24 | 29.328 | 0.088 |
| rs2024682 | 323 | 15.613 | 4 | 22 | 26.236 | 26.238 | 0.002 |
| rs5998714 | 149 | 15.4 | 3 | 22 | 31.784 | 31.792 | 0.008 |
| rs1018786 | 140 | 45.458 | 10 | 22 | 32.536 | 32.544 | 0.008 |
| rs1014208 | 108 | 24 | 4 | 22 | 42.384 | 42.395 | 0.011 |
| rs1076917 | 255 | 31.076 | 9 | 22 | 46.162 | 48.207 | 2.045 |
| rs738738 | 225 | 13.397 | 3 | 22 | 46.501 | 46.513 | 0.012 |

Table S2. Poisson analysis for 368 independent deletions (361 transmitted plus 7 *de novo* deletions) distributed over 4490 units of 0.6 Mb (based on 2694 Mb of autosomal DNA covered by the 317K Illumina platform). Clustering was calculated by subtracting the distance of each deletion starting point from that of the next starting point and grouping together those consecutive distances that total less than 0.6 Mb.

| k value | P value | number expected | number observed |
| --- | --- | --- | --- |
| 0 | 0.9213 | 4137 | 4194 |
| 1 | 0.0755 | 339 | 249 |
| 2 | 0.00309 | 13.9 | 33 |
| 3 | 0.0000844 | 0.38 | 6 |
| 4 | 0.00000173 | 0.008 | 5 |
| 5 | 2.83E-08 | 0.00013 | 1 |
| 8 | 4.64E-14 | 2E-10 | 1 |

P=λ^k^ e^(-λ)^/k!; λ=368/4490; e^(-λ)^=0.9213

Table S3 - Primers used for rare deletion validation by qPCR

| Primer ID | Sequence | Position (May 2004 assembly) |
| --- | --- | --- |
| R1-C1-01F | attcactttcttcaacagggcttt | chr02: 50691422-50691445 |
| R1-C1-01R | gtctttcatctctttgcctaaatgg | chr02: 50691671-50691647 |
| R1-T2-02F | ccttcctttttctctccctttctgc | chr02: 50741387-50741411 |
| R1-T2-02R | aagccatccctgtagttggtcctat | chr02: 50741657-50741633 |
| R1-T3-02F | atcgggtcttcagcaaaggtg | chr02: 50758723-50758743 |
| R1-T3-02R | tggatgggattcttaccacaacg | chr02: 50758959-50758937 |
| R1-T5-04F | tatgcccctgctcaagtatg | chr02: 50761935-50761954 |
| R1-T5-04R | gtaactggctttgtttctcgctat | chr02: 50762079-50762056 |
| R1-T6-01F | aactaaatgaaaatagcccagtgat | chr02: 50813647-50813671 |
| R1-T6-01R | taagccacagagaacaacaccaag | chr02: 50813812-50813789 |
| R1-T8-02F | cgatgaagagcgtggtgttgc | chr02: 51166672-51166692 |
| R1-T8-02R | ctgctgcgagagcgagatgag | chr02: 51166899-51166919 |
| H6PD-F | TCTTCATCACCACAGAGAACTTGC | chr01: 9258233-9258256 |
| H6PD-R | GACCTGGAAGTCACTGGGCA | chr01: 9258453-9258434 |
